# Transcriptional profiling of *Medicago truncatula *under salt stress identified a novel CBF transcription factor MtCBF4 that plays an important role in abiotic stress responses

**DOI:** 10.1186/1471-2229-11-109

**Published:** 2011-07-01

**Authors:** Daofeng Li, Yunqin Zhang, Xiaona Hu, Xiaoye Shen, Lei Ma, Zhen Su, Tao Wang, Jiangli Dong

**Affiliations:** 1 State Key Laboratory of Agrobiotechnology, College of Biological Sciences, China Agricultural University, Beijing, 100193, China; 2 State Key Laboratory of Plant Physiology and Biochemistry, College of Biological Sciences, China Agricultural University, Beijing 100193, China

## Abstract

**Background:**

Salt stress hinders the growth of plants and reduces crop production worldwide. However, different plant species might possess different adaptive mechanisms to mitigate salt stress. We conducted a detailed pathway analysis of transcriptional dynamics in the roots of *Medicago truncatula *seedlings under salt stress and selected a transcription factor gene, *MtCBF4*, for experimental validation.

**Results:**

A microarray experiment was conducted using root samples collected 6, 24, and 48 h after application of 180 mM NaCl. Analysis of 11 statistically significant expression profiles revealed different behaviors between primary and secondary metabolism pathways in response to external stress. Secondary metabolism that helps to maintain osmotic balance was induced. One of the highly induced transcription factor genes was successfully cloned, and was named *MtCBF4*. Phylogenetic analysis revealed that MtCBF4, which belongs to the AP2-EREBP transcription factor family, is a novel member of the CBF transcription factor in *M. truncatula*. MtCBF4 is shown to be a nuclear-localized protein. Expression of *MtCBF4 *in *M. truncatula *was induced by most of the abiotic stresses, including salt, drought, cold, and abscisic acid, suggesting crosstalk between these abiotic stresses. Transgenic *Arabidopsis *over-expressing *MtCBF4 *enhanced tolerance to drought and salt stress, and activated expression of downstream genes that contain DRE elements. Over-expression of *MtCBF4 *in *M. truncatula *also enhanced salt tolerance and induced expression level of corresponding downstream genes.

**Conclusion:**

Comprehensive transcriptomic analysis revealed complex mechanisms exist in plants in response to salt stress. The novel transcription factor gene *MtCBF4 *identified here played an important role in response to abiotic stresses, indicating that it might be a good candidate gene for genetic improvement to produce stress-tolerant plants.

## Background

Salt stress has a major effect on food production and quality worldwide by limiting the growth, development, and yield of crops [1]. More than one-fifth of the world's arable land is now under the threat of salt stress. As the global population increases, water resource management is deteriorating and environmental pollution is worsening; salinization of land is becoming more extreme and has begun to hinder development of agricultural economics.

Salt stress can damage plants by several mechanisms, including water deficit, ion toxicity, nutrient imbalance, and oxidative stress [2]. Plants respond and adapt to salt stress through a series of biochemical and physiological changes, involving expression and coordination of many genes [3,4]. Gene expression in the model plant *Arabidopsis thaliana *in response to salt and other abiotic stresses has been studied extensively [5,6]. However, conclusions derived from research conducted on *Arabidopsis *may not be applicable to other species, so research on species-specific responses to a particular abiotic stress is needed. Fabaceae is the third-largest family of flowering plants in the world, and contains many important crops that provide humans and animals with proteins [7]. Legumes are also important sources of edible oil and industrial fuel. *Medicago truncatula *is used as a model legume plant because of features such as a relatively small genome, self-pollination, a short life cycle, and the ability to form root nodules in association with rhizobia [8,9].

High-throughput expression profiling, such as microarray technology, has been used widely to study abiotic-stress-responsive mechanisms in plants. Transcriptional profiling of chickpea using a cDNA microarray revealed that 109, 210, and 386 genes were differentially regulated after drought, cold, and high-salinity treatment, respectively [10]. The iron-stress response of two near-isogenic soybean (*Glycine max*) lines was monitored using the Affymetrix GeneChip Soybean Genome Array, which indicated a transcription factor mutation appears to cause iron use inefficiency in soybeans [11]. Many genes of transgenic *Arabidopsis *over-expressing pea ABR17 (abscisic acid-responsive protein) exhibited different expression patterns under salt stress compared to the wild type, based on a 70-mer oligonucleotide probe microarray, indicating that ABR17 plays a role in mediating stress tolerance [12]. Another salt-stress response study of the root apex of *M. truncatula *using the Mt16K+ microarray identified 84 transcription factors exhibiting significant expression changes; some of these transcription factors belong to the AP2/EREBP and MYB transcription factor family [13]. Based on microarray analysis, genes involved in abiotic stress responses have been identified and categorized into two types according to the protein they code for [14]. The first type of genes expressed functional proteins, such as water-channel protein and membrane transporter protein; the second type was involved in signal transduction and expression regulatory processes, such as transcription factors and kinases [4]. Transcription factors could bind to the *cis*-elements of many target genes and regulate their expression, so these are good candidates for transgenic research to improve salt resistance among crops [5,15].

The AP2/EREBP family, which comprises a large group of transcription factors, plays functionally important roles in plant growth and development, especially in hormonal regulation and response to biotic and abiotic stress [16]. In *Arabidopsis*, 145 AP2/EREBP transcription factors were classified into five subfamilies, including DREB/CBF (dehydration-responsive element-binding protein/C-repeat binding factor), ERF (ethylene-responsive transcription factor), AP2 (APETALA 2), RAV (related to ABI3/VP1), and one specific gene, AL079349, based on similarities in their DNA-binding domain (AP2/ERF domain) [17]. The *Arabidopsis *genome contains six *DREB1/CBF *and eight *DREB2 *genes [18]. *DREB1A/CBF3, DREB1B/CBF1*, and *DREB1C/CBF2 *appear to be rapidly and transiently induced by cold; they are major transcription factors required for the expression of cold-inducible genes [19]. *DREB2A *and *DREB2B *genes are induced by dehydration and high salinity but not by cold stress, and they play very important roles in the osmotic stress response [19,20]. Expression of *DREB1D/CBF4 *is up-regulated by abscisic acid (ABA), drought, salt, and cold stress [18,21,22]. *AtDREB1E/DDF2 *(DWARF AND DELAYED FLOWERING 2) and *AtDREB1F/DDF1*, encoding another two AP2 transcription factors of the DREB1/CBF subfamily, are induced by high-salinity stress [23]. Over-expression of the *AtDREB1F/DDF1 *gene causes dwarfism and higher tolerance to salt stress, mainly by reducing levels of bioactive gibberellins (GA) in transgenic *Arabidopsis *[24]. Transgenic plants over-expressing *AtDREB1E/DDF2 *share a similar phenotype [24]. *DREB2C*, another member of the DREB2 transcription factor family, is induced by salt and cold, and transgenic plants over-expressing *DREB2C *become ABA hypersensitive [25]. The signal-transduction pathways in response to abiotic stress are complex, and the exact mechanisms require further study.

Recent research has isolated many DREB transcription factors from soybean, an important Fabaceae member, including *GmDREBa, GmDREBb, GmDREBc, GmDREB1, GmDREB2*, and *GmDREB3*. Expression of *GmDREBa *and *GmDREBb *is induced by salt, drought, and cold stress in the leaves of seedlings. By contrast, transcription levels of *GmDREBc *are apparently induced in roots by salt, drought, and ABA treatments, but are not significantly affected in leaves [26]. These results indicate that these three genes function differently in response to abiotic stresses in soybean. *GmDREB2 *is induced by cold, salt, drought stress, and ABA treatment, conferring tolerance to drought and high-salinity stress in transgenic plants [27]. In contrast, *GmDREB3 *is induced by cold stress only, and transgenic *Arabidopsis *are more tolerant to freezing, salt, and drought stress [28].

*MtCBF1, MtCBF2, MtDREB1C/CBF3*, and *MtDREB2A *have been identified in *M. truncatula*. The expression levels of *MtCBF2 *and *MtCBF3 *increased during treatment at 8°C and 6°C [29]. Over-expression of *MtCBF3/DREB1C *suppressed shoot growth and enhanced the freezing tolerance of transgenic *M. truncatula *[30]. This finding indicates that *MtCBF2 *and *MtCBF3/DREB1C *may play a critical role in inducing the *COLD-ACCLIMATION-SPECIFIC *(*CAS*) gene, which in turn increases freezing tolerance. In contrast, transcription levels of *MtDREB2A *are significantly up-regulated in roots by salt and drought stress treatments. Transgenic *M. truncatula MtDREB2a *(a constitutively active form of *MtDREB2A*) plants exhibit significantly dwarfed seedlings [28], but the stress tolerance of transgenic lines requires further study. Moreover, the relationships among the *MtDREB/CBFs *genes are still unknown.

*Agrobacterium rhizogenes*-mediated transformation of roots as well as transient transfection by vacuum infiltration of intact leaves is widely used in function analysis in legumes [31-33]. The transient transformation methods are faster and easier compared with the stable transformation technique.

In previous research, we constructed an expression database for *M. truncatula *under salt stress [34]. In this study, we implemented a more detailed pathway analysis of transcriptomics dynamics. In addition, we identified a novel transcription factor gene, *MtCBF4*, which was induced by salt, drought, cold, and abscisic acid. Over-expression of *MtCBF4 *in transgenic *Arabidopsis *and *M. truncatula *improved tolerance to abiotic stress and activated expression of downstream genes containing DRE elements, indicating that *MtCBF4 *might be a good candidate gene for improving the stress tolerance of transgenic plants.

## Results

### Root length measurement, selection of time-points and salt concentration

**Figure 1:**
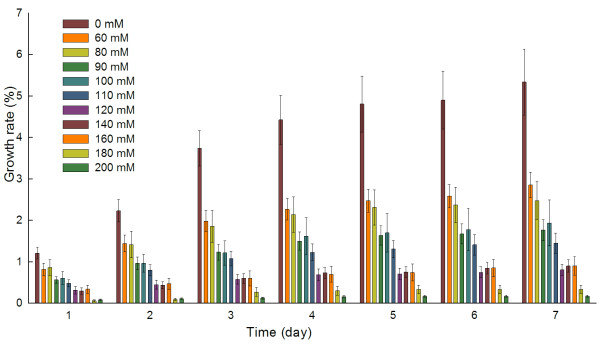
**Root growth rate of *M. truncatula *seedlings in response to different salt concentrations**. The data represent the daily percentage increase in root length over the seven-day treatment period. The raw data is available in Additional file 1.

We evaluated the root growth status of *M. truncatula *by measuring root lengths under different salt stress intensities at different time-points. Measurements were recorded at 12:00 pm every day for 1 week. Raw data for root length, calculated average length, growth rate and standard error values are presented in Additional file 1. The percentage increase in root length per day under different salt concentrations was calculated as a measure of growth rate (Figure 1). Roots treated with distilled water were used as the control (CK sample). The difference between a root growth rate and the value one day prior was tested using Student's *t*-test.

The growth rate of roots subjected to 180 mM NaCl stress was significantly different (p-value ≤ 0.05) after one day of treatment in contrast to other salt concentrations. Subsequently, roots treated with 60 mM and 120 mM NaCl also showed significantly different growth rates (Additional file 1, *t*-test based on NaCl concentration table). According to the *t*-test results, NaCl concentrations could be divided into three groups: 60~110 mM 120~160 mM, and 180~200 mM, corresponding to low, intermediate and high levels of salt stress, respectively. In order to induce a higher frequency of expression changes and maintain seedling activity simultaneously, as some seedlings under a high level of salt stress ceased growth from the third day of stress treatment, we chose 180 mM NaCl and selected days 1 and 2 as time-points to investigate the effects of a high level of salt stress on *M. truncatula *seedlings. In addition, 6 h post-treatment was added as a time-point to examine the early stages of the stress response. The I_50 _value is defined as the concentration of NaCl that reduces the rate of root growth by 50% relative to a control; this value was used to measure the salt tolerance level. The I_50 _of *Arabidopsis *is about 100 mM NaCl [35]. Our salt concentration gradient tests revealed that the I_50 _of *M. truncatula *Jemalong A17 was also about 100 mM based on root lengths measured after one day of salt stress (Additional file 1, I_50 _estimation table), which indicated the salt sensitivity of *M. truncatula*.

### Quantitative real-time PCR validation of microarray experiments

Microarray experiment design and related protocols were described in our previous work [34]. To validate the microarray results, five probe sets were selected to confirm expression differences with quantitative real-time PCR (qRT-PCR): a MYB transcription factor (Mtr.15010.1.S1_S_at), a 2OG-Fe(II) oxygenase family protein (Mtr.40379.1.S1_at), a delta l-pyrroline-5-carboxylate synthetase (Mtr.42902.1.S1_s_at), an AP2-EREBP transcription factor (Mtr.38878.1.S1_at), and a dehydrin-like protein (Mtr.8651.1.S1_at). Selection of these probe sets was based on their statistically significant up-regulated expression and abundant annotations. 2OG-Fe(II) oxygenase is reported to be involved in plant defense [36], and l-pyrroline-5-carboxylate synthetase plays a role in the drought stress response [37]. Several dehydrin-like proteins show diverse accumulation in many plants in response to cold and heat stress [38]. Many members of the transcription factor family AP2-EREBP and MYB are reported to be involved in abiotic stress responses [39-41]. The qRT-PCR method is much more sensitive than microarray analysis for detecting transcript expression and can be used as a confirmatory tool for microarray results [42,43]. Although the quantitative values differed considerably, the same trend was observed in the qRT-PCR and microarray analyses (Additional file 2). The primers used are listed in Additional file 3 (Table S1), and *MtActin *[33] was used as a control.

### Gene expression profiles and pathway enrichment analysis

**Figure 2:**
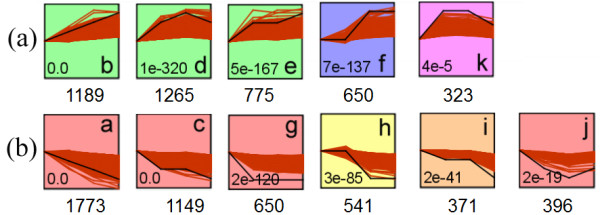
**Significant expression profiles**. Statistically significant pathways (*a *to *k*) are categorized into two groups: (a) up-regulated profiles (b) down-regulated profiles. Numbers of probe sets assigned to each profile are represented below. P-values are shown in left bottom of each profiles, only statistically significant (P-value < 0.05) expression profiles are shown (profiles were produced using STEM software).

We analyzed expression data for 50,900 *M. truncatula *probe sets at the three time-points using STEM software [44] to cluster the expression patterns. A total of 11 significant expression profiles were generated based on a p-value of 0.05. Based on the expression patterns, profiles were classified into two categories (Figure 2): those with an up-regulated pattern, including profile *b, d, e, f *and *k*, assigned with 1,189, 1,265, 775, 650, and 323 probe sets, respectively (Figure 2a); and those with a down-regulated pattern, including profile *a, c, g, h, i*, and *j *assigned with 1,773, 1,149, 650, 541, 371, and 396 probe sets, respectively (Figure 2b). Probe sets belonging to each profile were subjected to an enrichment analysis at the pathway level using the PathExpress tool [45,46], and significant pathway distributions for each profile were shown (Table 1). Probe sets related to primary metabolism (such as glycolysis, carbon fixation, starch, and sucrose metabolism) were mainly repressed in response to salt stress. Of all the probe sets that might be involved in the anthocyanin biosynthesis pathway, half of the probe sets had been up-regulated. The flavonoid biosynthesis pathway was suppressed, as more than half of the probe sets involved in this pathway were down-regulated. However, the six probe sets involved in the isoflavonoid biosynthesis pathway were all up-regulated: two isoflavone 7-O-methyltransferases (Mtr.37751.1.S1_at and Mtr.16873.1.S1_s_at) in profile *b; *an isoflavone 7-O-methyltransferase (Mtr.49862.1.S1_at) and an isoflavone 3'-hydroxylase (Mtr.12632.1.S1_at) in profile *d*; and two isoflavone reductases (Mtr.30508.1.S1_at and Mtr.410.1.S1_s_at) in profile *e*.

**Table 1:** Summary of Pathway Enrichment Analysis

Profile	Pathway	Nb. of Enzymes	Nb. of Enzymes submitted	P-value
*a*	Glycolysis/Gluconeogenesis	26	13	1.47e^-3^
	Starch and sucrose metabolism	33	15	2.15e^-3^
	Streptomycin biosynthesis	4	4	2.43e^-3^
	Biosynthesis of steroids	28	12	1.09e^-2^
	Ascorbate and aldarate metabolism	8	5	1.64e^-2^
	Pentose and glucuronate interconversions	12	6	3.16e^-2^
	Anthocyanin biosynthesis	2	2	4.98e^-2^
	1,2-Dichloroethane degradation	2	2	4.98e^-2^
	Flavonoid biosynthesis	10	5	4.98e^-2^
*b*	Starch and sucrose metabolism	33	9	4.51e^-3^
	Anthocyanin biosynthesis	2	2	1.09e^-2^
	Glycerolipid metabolism	17	5	2.51e^-2^
	Phenylpropanoid biosynthesis	7	3	2.85e^-2^
	Sphingolipid metabolism	7	3	2.85e^-2^
	Isoflavonoid biosynthesis	3	2	3.04e^-2^
	Valine, leucine and isoleucine degradation	18	5	3.19e^-2^
*c*	Starch and sucrose metabolism	33	13	9.17e^-4^
	Glycolysis/Gluconeogenesis	26	11	1.17e^-3^
	Carbon fixation	22	8	1.66e^-2^
	Valine, leucine and isoleucine biosynthesis	13	5	4.52e^-2^
	Pyrimidine metabolism	26	8	4.59e^-2^
*d*	Phenylpropanoid biosynthesis	7	6	3.30e^-6^
	Flavonoid biosynthesis	10	4	9.25e^-3^
	Isoflavonoid biosynthesis	3	2	2.39e^-2^
	Metabolism of xenobiotics by cytochrome	4	2	4.50e^-2^
*e*	Flavonoid biosynthesis	10	5	5.25e^-4^
	Anthocyanin biosynthesis	2	2	6.39e^-3^
	Phenylpropanoid biosynthesis	7	3	1.38e^-2^
	Isoflavonoid biosynthesis	3	2	1.82e^-2^
	Zeatin biosynthesis	4	2	3.45e^-2^
	Glycerolipid metabolism	17	4	4.08e^-2^
	Starch and sucrose metabolism	33	6	4.18e^-2^
	Methionine metabolism	18	4	4.94e^-2^
*f*	Galactose metabolism	18	5	4.30e^-3^
	Starch and sucrose metabolism	33	6	1.60e^-2^
	Glycosphingolipid biosynthesis - globoseries	5	2	3.69e^-2^
*g*	Flavonoid biosynthesis	10	4	5.92e^-3^
	Anthocyanin biosynthesis	2	2	6.64e^-3^
	Starch and sucrose metabolism	33	7	1.36e^-2^
	Phenylpropanoid biosynthesis	7	3	1.45e^-2^
	Aminoacyl-tRNA biosynthesis	21	5	2.29e^-2^
*h*	Starch and sucrose metabolism	33	10	5.11e^-4^
	Anthocyanin biosynthesis	2	2	9.06e^-3^
	Flavonoid biosynthesis	10	4	1.04e^-2^
	Drug metabolism - cytochrome P450	6	3	1.36e^-2^
	Phenylpropanoid biosynthesis	7	3	2.22e^-2^
	3-Chloroacrylic acid degradation	3	2	2.55e^-2^
	Glycolysis/Gluconeogenesis	26	6	3.04e^-2^
	Bile acid biosynthesis	4	2	4.79e^-2^
	Metabolism of xenobiotics by cytochrome P450	4	2	4.79e^-2^
	Streptomycin biosynthesis	4	2	4.79e^-2^
*i*	Pentose and glucuronate interconversions	12	3	1.30e^-2^
	Phosphatidylinositol signaling system	13	3	1.64e^-2^
	Pyrimidine metabolism	26	4	2.33e^-2^
	Inositol phosphate metabolism	15	3	2.46e^-2^
	Phenylpropanoid biosynthesis	7	2	3.44e^-2^
	Benzoxazinone biosynthesis	1	1	4.41e^-2^
*j*	Flavonoid biosynthesis	10	4	2.74e^-3^
	Phenylpropanoid biosynthesis	7	3	8.13e^-3^
	Indole and ipecac alkaloid biosynthesis	5	2	3.85e^-2^
*k*	Anthocyanin biosynthesis	2	2	2.76e^-3^
	Flavonoid biosynthesis	10	3	1.29e^-2^
	Drug metabolism - cytochrome P450	6	2	3.62e^-2^

Pathway enrichment analysis was done by PathExpress tool. Only statistic significant (p-value < = 0.05) pathways left. Profiles numbered from *a *to *k *were listed in Figure 2. The "No. of Enzymes" column means how many enzymes of each pathway are in the array, the "No. of Enzymes submitted" column means how many enzymes belong to each profile.

We utilized the GeneBins tool [47] for additional pathway analyses, because probe sets of *M. truncatula *were more annotated by GeneBins than PathExpress. We selected probe sets that were up- or down-regulated by more than two-fold at each time-point versus values at 0 h for GeneBins analysis and to assess their distribution on the STEM-generated profiles. Additional file 4 summarizes these probe sets and their corresponding profiles, along with the GeneBins functional annotation. Those probe sets were also functionally categorized using the GeneBins second-level ontology. Almost all probe sets belonging to profiles *a, c, g, h, i*, and *j *were classified as down-regulated, and almost all probe sets belonging to profiles *b, d, e, f*, and *k *were up-regulated. Unclassified (i.e., not annotated by GeneBins ontology) probe sets were not subjected to the analysis described below. Profile *a *displayed a continuing downward trend. Most probe sets in this profile were classified into the main metabolism category, and the number of probe sets increased with longer exposure to salt. This result might be caused by the inhibition of plant growth by salt stress, which causes water potential, osmotic, and nutritional imbalances [48]. Other subcategories of profile *a *contained a considerable proportion of probe sets including genes for translation, folding and sorting, degradation, and signal transduction. The translation process was repressed as probe sets assigned to this category were more down-regulated; this indicated that growth activity in seedlings was constrained by external stress at the translational level. The up-regulated profiles *b, d, e, f*, and *k *not only contained a considerable proportion of probe sets involved in primary metabolism processes (carbohydrate, lipid, and amino acid metabolism), but also in secondary metabolism and information processes. The pathways metabolism of cofactors and vitamins, biosynthesis of secondary metabolites, and biodegradation of xenobiotics all showed considerably more probe sets that were up-regulated than down-regulated. This indicates that the plants have a positive response mechanism against external harmful stress.

### A novel member of CBF transcription factor in *M. truncatula*

**Figure 3:**
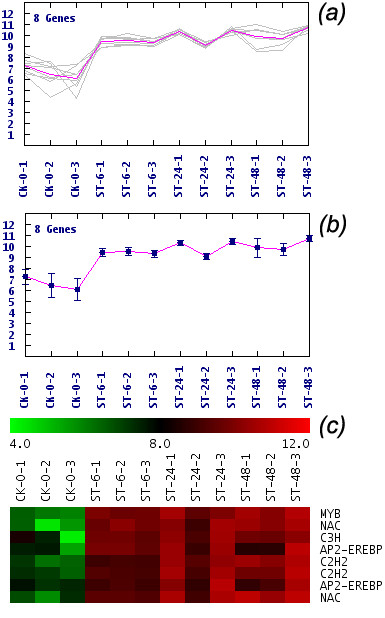
**Expression profile of *MtCBF4***. Expression profiles of 2138 transcription factors were re-clustered using the TIGR MeV tool. (a) Expression profile to which *MtCBF4 *belongs. The pink line represents the main trend line. (b) Euclidian distance map of this profile. (c) Heatmap display of this profile. The color scale bar represents log_2_-transformed expression values from 4 to 12. The label at the right of each row represents the transcription factor family to which the probe set belongs. CK, control sample; ST, salt-treated sample; 6, 24, 48 are the time-points for salt stress measurement; 1, 2, and 3 indicate three biological replicates.

We used probe consensus sequences of the array for a BLASTX search against the *M. truncatula *transcription factor (TF) peptide sequences database, and found 2138 probes that matched (i.e., showed sequence homology) the 1022 TFs obtained from PlantTFDB [49], excluding the probe sets of *M. sativa *and *Sinorhizobium meliloti*. The 2138 TFs were isolated for further cluster analysis using the MeV tool [50] based on their gene expression regulation and signal transduction role [15]. In the re-clustered results by expression profiles of the 2138 TFs (Additional file 5), eight TFs in one of the profiles that exhibited an up-regulating trend were selected for further analysis (Figure 3 and Additional file 5, indicated by star). Of the eight TFs, the probe set Mtr.38878.1.S1_at belonged to the AP2/EREBP transcription factor family and shares the most similarities with the CRT/DRE binging factor (CBF) as indicated by a BLAST search against the NCBI *nr *database. Seven other transcription factors belonged to the MYB, NAC, C3H, and C2H2 families. With the exception of the C3H family, members of these families are reportedly capable of abiotic stress responses [51-53]. As CBF TFs are reported to participate in many abiotic stress responses, Mtr.38878.1.S1_at was further chosen for function validation. We named this novel member of the AP2/EREBP transcription factor gene *MtCBF4*, because previous studies have identified and analyzed *MtCBF1, MtCBF2*, and *MtCBF3 *[29]. *MtCBF4 *contained an open reading frame of 618-bp, encoding a protein of 205 amino acids, with a predicted molecular mass of 23.1 kD and a pI of 5.1. The 618-bp sequence was submitted to GenBank (accession no. HQ110079.1). To date, four *CBF *genes of *Medicago *have been isolated (including *MtCBF4*), and two of these (*MtCBF2 *and *MtCBF3*) have been proven to play roles in the response to cold stress [29]. However, the relationships among them are still unknown.

To examine the phylogenetic relationship of the DREB/CBF family, we compared the amino acid sequence of MtCBF4 with 22 DREB/CBF family members from *Arabidopsis, Glycine max*, and *Medicago truncatula *(Additional file 6). The phylogenetic analysis revealed that DREB/CBF proteins were grouped by species, and all the CBFs were clustered together according to DREB type. DREB1-type CBFs were clustered together. MtCBF4 was most similar to GmCBF2 and GmCBF1, and in turn to MtCBF3 and MtCBF2. MtCBF4 showed higher similarity to AtDREB1/CBFs than to AtDREB2, therefore it was classified as a DREB1-type CBF. The amino acid sequence of MtCBF4 shared 57% (E-value = 1e^-46^) sequence identity against AtCBF4, which was higher than the identities between MtCBF4 and AtCBF1 (53%, E-value = 2e^-42^), AtCBF2 (53%, E-value = 1e^-43^) and AtCBF3 (53%, E-value = 6e^-47^), respectively.

**Figure 4:**
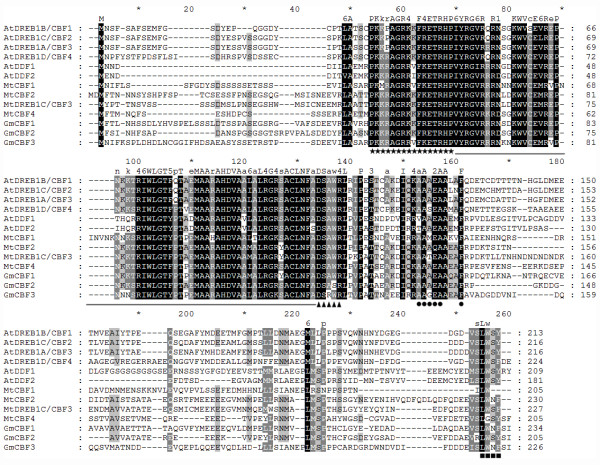
**Multiple sequence alignment of 13 DREB/CBF homologs**. Amino acid residues highlighted in black were conserved in more than half of the sequences; residues highlighted in gray share similar chemical properties. Amino acid positions and consensus sequences are shown at the top of each panel. The conserved AP2 DNA-binding domain is indicated as the underlined segment. Stars and triangles indicate the CBF signature sequences; squares indicate the LWSY domain; circles indicate the conserved motif among CBF homologs.

We also compared the amino acid sequence of MtCBF4 with several DREB-1 related proteins. As shown in Figure 4, MtCBF4 protein had a conserved AP2 DNA-binding domain similar to other CBF proteins. The CBF signature sequences (PKK/RPAGRxKFxETRHP and DSAWR, located immediately before and after the AP2 domain, respectively; A(A/V)xxA(A/V)xxF, with the underlined residues conserved in all known CBF homologs, located downstream of the DSAWR) [54,55] as well as the C-terminal LWSY motif [56] were also conserved in the MtCBF4 protein. The MtCBF4 protein contains DSAWK instead of DSAWR. As mentioned above, the amino acid sequence of the MtCBF4 protein shared 57% similarity with the *Arabidopsis *CBF4 protein (AtDREB1D/CBF4), indicating *MtCBF4 *is a homolog of *AtDREB1D/CBF4*.

We also checked the promoter sequence (1000 bp upstream from the translation start site) of *MtCBF4 *using the PLACE Signal Scan Search Program [57]. The promoter sequence contained many putative stress-responsive *cis*-elements such as ABRE (the core sequence of ABRE), and recognition sites for MYB, MYC and WRKY transcription factors (Additional file 7). These *cis*-elements (ABRE, MYBRS and MYCRS) and the corresponding transcription factors (AREB/ARF, MYB and MYC transcription factors) play important roles in the ABA signaling pathway and abiotic stress responses [15,58]. WRKY transcription factors are suggested to be involved in response and adaptation to abiotic and/or biotic stresses [59,60].

### Localization and transactivation of MtCBF4 protein

**Figure 5:**
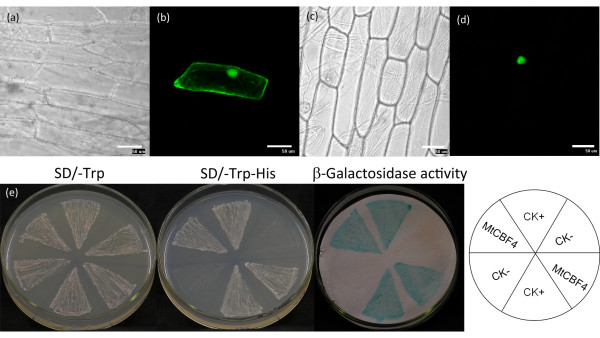
**Subcellular localization and transcriptional activation analysis of MtCBF4**. *MtCBF4:GFP *was bombarded into onion epidermal cells with DNA-coated gold particles, and GFP expression was visualized after 16 h. Cells expressing GFP were used as a control. Images represent GFP alone (b) and MtCBF4-GFP (d) in onion epidermal cells with corresponding bright-field images (a and c). Growth of pBD GAL4-MtCBF4 and pGAL4 transformants on SD/-Trp-His medium and the blue color in the β-galactosidase assay indicated MtCBF4 exhibits transactivation activity (e). The pBD GAL4 empty vector was used as the negative control, and pGAL4 vector was used as the positive control. All of the transformants grew well on SD/-Trp medium. Bars = 50 μm.

To determine its subcellular localization, *MtCBF4 *was fused in frame to the 5' terminus of the green fluorescent protein (GFP) reporter gene under the control of the cauliflower mosaic virus dual 35S promoter (CaMV 35S), as well as a tobacco etch virus (TEV) enhancer. The recombinant constructs of the *MtCBF4-GFP *fusion gene and *GFP *alone were introduced into onion (*Allium cepa*) epidermal cells via a gene gun (Bio-Rad, California, USA). The MtCBF4-GFP fusion protein accumulated mainly in the nucleus, whereas GFP alone was present throughout the whole cell (Figure 5a-d). Thus, MtCBF4 was a nuclear-localized protein, which was consistent with its predicted function as a transcription factor.

The transactivation ability of MtCBF4 was analyzed using a yeast assay system. The GAL4 DNA-binding domain-*MtCBF4 *recombinant plasmid was transformed into yeast cells and assayed for its ability to activate transcription of the dual report genes *His3 *and *LacZ *both controlled by the GAL4 upstream activation sequence. Yeast cells with the fusion plasmids harboring *MtCBF4 *grew on SD medium lacking histidine, and were stained blue in X-Gal solution (Figure 5e). These results indicated that MtCBF4 showed transactivation capability.

### Expression pattern of *MtCBF4 *under different abiotic stresses

**Figure 6:**
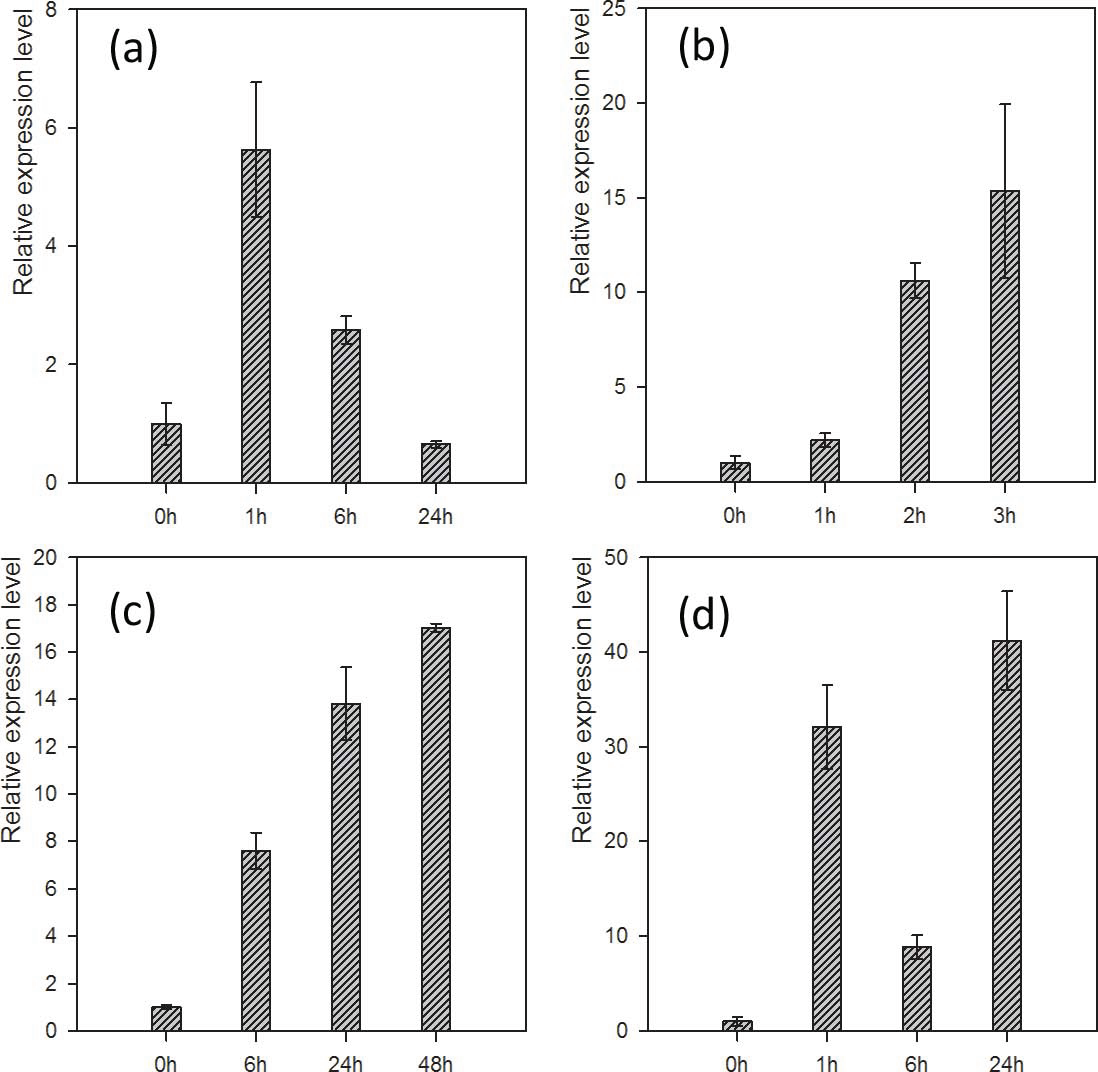
**Expression of *MtCBF4 *in response to ABA, drought, salt and cold treatments**. Four-week-old seedlings were subjected to the following treatments: (a) 200 μl ABA solution containing 0.05% Tween20 (v/v) was sprayed onto leaves for 1, 6, or 24 h; (b) For drought treatment, seedlings were transferred to dry Whatman 3 MM paper in a sterile Petri dish for 1, 2, or 3 h; (c) Seedlings were treated for 6, 24, or 48 h with 180 mM NaCl; (d) Seedlings were placed in a growth chamber at 4°C for 1, 6, or 24 h. The *MtActin *gene was amplified as a control. Data represent the mean and standard error (SE) for three replications. Primers used are listed in Additional file 3 (Table S1).

We conducted a qRT-PCR to examine the expression pattern of *MtCBF4 *under different stress conditions. At 1 h after ABA treatment, the transcript level of *MtCBF4 *had increased almost six-fold; thereafter, it decreased to the pretreatment level after 24 h (Figure 6a). Under drought stress, the transcription level of *MtCBF4 *began to increase within 1 h and continued to increase after 3 h (Figure 6b). With regard to salt stress, the transcription level of *MtCBF4 *began to increase at 6 h and continued to increase after 48 h (Figure 6c). Treatment with cold stress yielded very interesting results; the transcription level of *MtCBF4 *rose sharply within 1 h after treatment compared to that of the non-treated control, then fell sharply but remained above the pretreatment level at 6 h, and then rose again to a much higher level at 24 h (Figure 6d). Taken together, these results reveal that *MtCBF4 *was induced by ABA, drought, salt, and cold stimulation, indicating that it might play an important role in response to abiotic stresses and ABA treatment.

### Over-expression of *MtCBF4 *improved drought and high-salinity tolerance in transgenic *Arabidopsis*

**Figure 7:**
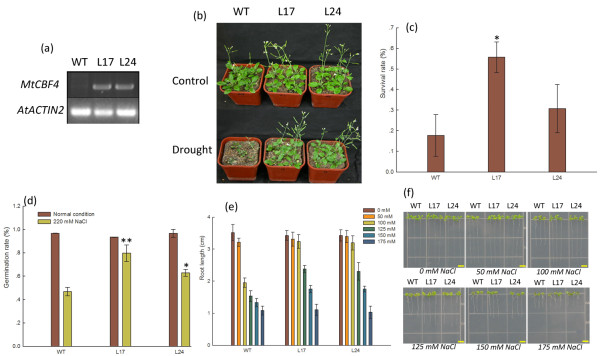
**Analysis of *35S:MtCBF4 *transgenic *Arabidopsis *lines**. (a) RT-PCR analysis of *Arabidopsis 35S:MtCBF4 *transgenic lines (L17 and L24) and the wild type (WT). (b) Comparison of plants subjected to 16 d drought stress treatment and control plants. (c) Percentage survival of L17 and L24 plants exposed to 16 d drought stress. Mean survival and standard deviation (SD) were calculated from the results of three replicated experiments each using more than 60 seedlings. Asterisk indicates that these plants had significantly higher survival rates under drought treatment than wild-type plants (Student's *t*-test, *P < 0.05). (d) Germination of L17 and L24 seeds in the presence of 220 mM NaCl. Mean germination and SD were calculated from the results of three replicated experiments each using more than 60 seeds. Asterisk indicates that these plants had significantly higher germination rates under 220 mM NaCl than wild-type plants (Student's *t*-test, **P < 0.01, *P < 0.05). (e) Root growth of L17 and L24 seedlings in the presence of different concentrations of NaCl. Three days after germination on MS agar plates, WT and transgenic seedlings were transferred to a new MS agar plate containing different concentrations of NaCl for 7 days. The seedling root lengths were measured with Image software. (f) Root lengths of the means of three replicated experiments. Error bars indicate SD (n = 18). Bars = 0.5 cm.

The notable induction of *MtCBF4 *expression by multiple stresses indicated this gene might be involved in stress resistance. Expression of *MtCBF4 *in transgenic *Arabidopsis *was detected by RT-PCR (Figure 7a). We randomly selected two independent T_3 _*MtCBF4 *over-expressing lines (L17 and L24) for drought and salinity resistance testing. Over-expression of *MtCBF4 *in both lines did not cause significant growth retardation compared with the wild type as indicated by inflorescence height and seed yield per plant (Additional file 8).

Three-week-old seedlings were used for drought tolerance assays. After 16 days without water, all pots were watered simultaneously and plant recovery and survival rate were recorded. T_3 _transgenic *Arabidopsis *plants over-expressing *MtCBF4 *showed enhanced drought tolerance, as wild-type plants had wilted compared to transgenic lines L17 and L24 after drought treatment (Figure 7b). Overall, 17.71% (34/192) of wild-type plants survived, whereas the survival rates of the *35S:MtCBF4 *L17 and L24 transgenic plants were 55.68% (103/185; P-value = 0.02, *t *test) and 30.73% (55/179; P-value = 0.324, *t *test), respectively (Figure 7c).

We tested the effect of NaCl on germination of *MtCBF4*-over-expressing seeds. Seed germination of the wild-type and transgenic plants did not differ under normal conditions. However, in the presence of 220 mM NaCl seed germination differed significantly: 46.83% (92/229) of the wild-type seeds germinated, whereas the germination rates of the *35S:MtCBF4 *L17 and L24 transgenic lines were 79.66% (156/203, **p < 0.01, *t *test) and 62.86% (138/218, *p < 0.05, *t *test), respectively (Figure 7d).

To determine the effect of *MtCBF4 *over-expression on post-germination salt tolerance, 3 d after germination transgenic and wild-type seedlings were carefully transferred to new plates containing different concentrations of NaCl. At a NaCl concentration of 50 to 150 mM, seedlings of both transgenic lines displayed better root growth than the wild type. However, at 175 mM NaCl root growth was seriously inhibited and no significant difference was detected (Figure 7e, f). The I_50 _of the *MtCBF4 *transgenic plants was 150 mM NaCl, which exceeded that of wild-type *Arabidopsis *(about 100 mM NaCl). The results indicated over-expression of *MtCBF4 *in *Arabidopsis *increased salt tolerance during both germination and early seedling growth.

Since over-expression of *MtCBF4 *enhanced drought and salt stress tolerance in transgenic *Arabidopsis*, we examined the changes in expression of abiotic stress

**Figure 8:**
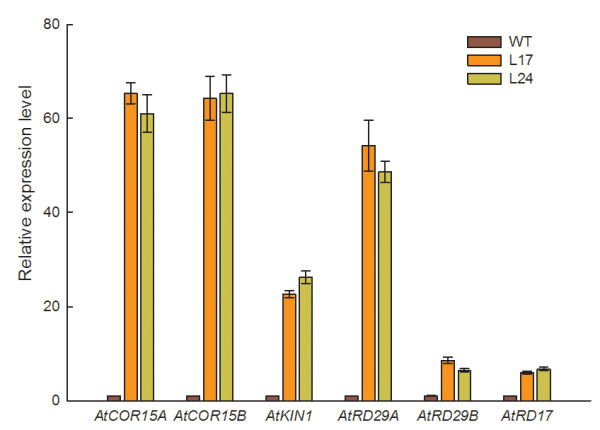
**Expression analysis of genes downstream of *MtCBF4 *in *Arabidopsis***. Abiotic stress-responsive genes in *MtCBF4 *transgenic and wild-type plants were analyzed by qRT-PCR. Total RNA was extracted from three-week-old seedlings grown under normal conditions. The graphs indicate the induction levels of *AtCOR15A, AtCOR15B, AtKIN1, AtRD29A, AtRD29B *and *AtRD17 *in the transgenic lines L17 and L24 compared with those of wild-type plants (WT). *AtACTIN *and *Atβ-TUBULIN *were amplified as controls. Data represent means and SE of three replications. Primers used are listed in Additional file 3 (Table S2).

-responsive genes in these plants. Six genes (*COR15A, COR15B, KIN1, RD17, RD29A*, and *RD29B*) that contain DRE elements in their promoter regions and have been identified as downstream genes of AtDREBs in *Arabidopsis *[61-63] were chosen for study. Total RNAs isolated from three-week-old wild-type, L17, and L24 seedlings were used for qRT-PCR analysis. Expression levels of all six genes were enhanced in *MtCBF4 *transgenic plants under normal growth conditions (Figure 8). These results indicated *MtCBF4 *up-regulated expression of downstream genes related to drought and salt stress responses.

### Over-expression of *MtCBF4 *enhances salt tolerance and induces two putative target genes in transient transgenic *M. truncatula*

**Figure 9:**
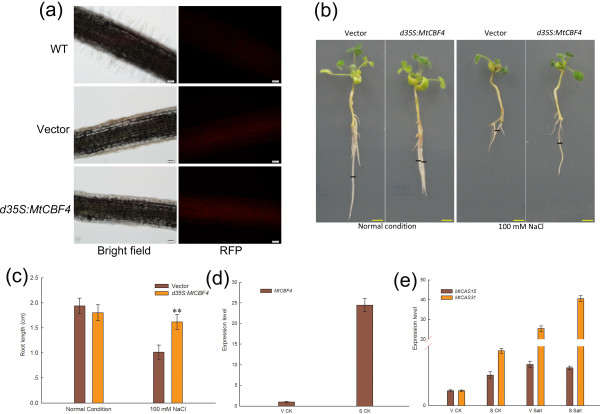
**Expression of *MtCBF4 *improved salt tolerance and activated two CBF target genes in *M. truncatula***. (a) RFP fluorescence in *A. rhizogenes*-transformed *M. truncatula *roots was observed with fluorescence microscopy 2 weeks after inoculation. In empty vector as well as *d35S:MtCBF4 *transgenic composite plants, RFP was observed in corresponding bright-field images (left). Wild-type plants were used as a control. Bars = 200 μm. (b) Representative examples of *MtCBF4*-overexpressing *A. rhizogenes*-transformed roots 1 week after transfer to control medium (left) or medium containing 100 mM NaCl (right). Black lines indicate the position of root tips at the moment of transfer. An empty pRedRoot vector was used as a control. Bars = 0.5 cm. (c) Primary root length of transgenic roots was measured from the point of transfer to salt-containing medium (100 mM NaCl) or normal medium after 1 week. A representative example of three replications is shown (n > 30 per construct and condition per experiment). Asterisk indicates that these plants had significantly longer root length under 100 mM NaCl than control plants (Student's *t*-test, **P < 0.01). (d) and (e) Expression of *MtCBF4 *and potential targets in transiently transfected *M. truncatula *leaves. RNA from leaves transformed with an empty vector (V) or *d35S:MtCBF4 *construct (S) were used for qRT-PCR after 48 h of transfection. For salt treatment, the leaves were treated with 100 mM NaCl for 6 h (Salt), and ddH_2_O treatment was used as a control (CK). Histograms show relative quantification of the transgene and the putative targets (*MtCAS15 *and *MtCAS31*). Data represent means and SE of three replications.

To investigate the putative role of *MtCBF4 *in response to salt stress, we prepared transgenic composite *M. truncatula *Jemalong A17 plants carrying *A. rhizogenes*-transformed roots over-expressing *MtCBF4 *[64]. The transgenic plants were identified by RFP detection (Figure 9a). Three weeks after inoculation, the seedlings were transferred to a new plate with salt-containing medium (100 mM NaCl in Fahraeus medium), and the root length was measured after one week. Under normal conditions, there was no significant difference in growth between over-expressing and control plants. However, a significant increase (Student's *t*-test, P-value = 0.007) in primary roots growth in the *MtCBF4*-overexpressing lines compared with the control plants was detected on the salt-containing medium (Figure 9b, c). Another two representative cultivars of *MtCBF4*-overexpressing *A. rhizogenes*-transformed *M. truncatula *roots were also presented in Additional file 9.

Further evidence was obtained by transient transfection of the leaves by vacuum infiltration [32]. *MtCAS15 *and *MtCAS31 *(GenBank accession nos. EU139869.1 and EU139871.1) belong to the CBF regulon [65], so we detected the expression level of these two targets in *d35S:MtCBF4 *transgenic *M. truncatula*. Under normal conditions, the induction rates of the two genes were about two-fold and three-fold, respectively, in *d35S:MtCBF4 *transgenic plants compared with the control plants (empty vector transformed plants). However, under salt treatment, the expression level of *MtCAS31 *increased 25-fold in control plants, whereas in *d35S:MtCBF4 *transgenic plants the expression level increased 40-fold. Expression of *MtCAS15 *did not differ significantly between the *d35S:MtCBF4 *transgenic and control plants under salt treatment (Figure 9d, e). Primers used are listed in Additional file 3 (Table S1 and Table S3). Overall, the results indicated that over-expression of *MtCBF4 *enhanced tolerance to salt stress and up-regulated the expression of downstream genes in *M. truncatula*.

## Discussion

### Pathway analysis of transcriptomic response to salt stress in *M. truncatula*

Plants produce many secondary metabolites that not only counteract environmental stress, but also aid growth and development [66]. It is possible that an increased rate of anthocyanin biosynthesis improves a plant's ability to guard against oxidative damage and protects it from such injury. Anthocyanin reportedly functions in the cell membrane as an antioxidant to prevent lipid peroxidation under conditions of stress [67,68]. Isoflavonoid reductase is a key enzyme in the isoflavonoid phytoalexin biosynthesis pathway and was first studied in *Medicago sativa *[69]. Over-expression of an isoflavone reductase-like gene in transgenic rice (*Oryza sativa*) reportedly confers resistance to reactive oxygen species (ROS) stress [70], which often occurs after ion stress caused by salt stress. Isoflavonoids are required for a wide range of essential physiological processes and are valuable secondary phenylpropanoid metabolites found mainly in legumes. Over-expression of isoflavone 7-O-methyltransferase reportedly increases disease resistance in *M. sativa *and is regarded as the entry point of the isoflavone pathway [71]. The isoflavone 3'-hydroxylase belongs to the cytochrome P450 81E family in *M. truncatula *and possesses many biotic defense responses [72]. The hydroxylation process depends on cytochrome P450 monooxygenases, which are critical to the isoflavone pathway [73], and the probe set Mtr.12632.1.S1_at, appears to be most similar to CYP81D8 (AT4G37370.1) in *Arabidopsis*. A recent study revealed that a flavonoid-deficient root of *M. truncatula *lost nodulation capacity, while an isoflavone-deficient root remained unaffected [74], indicating that the flavone and isoflavone pathways not only function differently during the nodulation process, but also during the response to salt stress. The mechanisms of the isoflavone biosynthesis pathway have been studied using *M. truncatula *as a model system, which demonstrated that it has tissue- and stress-specific expression patterns [75]; our findings confirmed its capability to respond to salt stress.

The active metabolism of cofactors and vitamins may help to establish the translation process; the biosynthesis of the secondary metabolites discussed above might help to re-establish the osmotic balance and reduce the threat of ROS, and the biodegradation of xenobiotics might help remove injurants generated or introduced by salt stress. Plants might require these response mechanisms against abiotic stress to survive. The response of plants to salt stress is very complex; this may be illustrated by the considerably higher number of probe sets up-regulated in signal transduction and ligand-receptor interaction pathways. Probe sets involved in protein folding, sorting, and degradation include heat shock proteins. It is worth noting that the probe sets related to cell growth and death processes showed greater up-regulation. Many studies have shown that abiotic stresses, such as salt stress, induce programmed cell death and also result in morphological, physiological, and biochemical changes in plants [76,77].

### A novel CBF member from *M. truncatula*

The DREB subfamily pathway plays an important role in the stress-responsive regulatory network in plants [17]. Currently, many homologous DREB genes have been identified in a variety of plants, such as *Arabidopsis*, rice, soybean, barley (*Hordeum vulgare*), cotton (*Gossypium hirsutum*), tomato (*Solanum lycopersicum*), tobacco (*Nicotiana tabacum*), and maize (*Zea mays*), and over-expression of these genes increased the tolerance to abiotic stresses [17-21,24,56,78-83]. Three *MtCBFs *(*MtCBF1, MtCBF2*, and *MtCBF3*) have been isolated, which were thought to play an important role in response to cold stress [29]. *MtCBF1 *was slightly induced by salt stress in our microarray data, while *MtCBF2 *and *MtCBF3 *showed no significant changes by checking their corresponding homologous probe sets. The novel CBF subfamily member *MtCBF4*, which encodes a homolog of CBF proteins in *M. truncatula*, showed the highest change in induced expression under salt stress, and was isolated in this study.

Tissue specificity analysis with the *Medicago truncatula *Gene Expression Atlas [84] indicated that *MtCBF4 *showed a relatively higher expression level in the stem, root (especially the root tip) and seed coat than in other tissues. *MtCBF1 *showed higher expression in the root and leaf, *MtCBF2 *was expressed more highly in the shoot and stem, and expression of *MtCBF3 *was higher in the nodule and root. Expression of *MtCBF4 *in the root and root tip indicated its involvement in the salt stress response, as the root is the first plant organ to perceive external stress in soil.

Phylogenetic analysis revealed MtCBF4 belonged to the DREB1-type class and was most similar to GmCBF2 and GmCBF1. However, the study of GmCBF2 and GmCBF1 is very limited. The amino acid sequence of the MtCBF4 protein also shares relatively high similarity (57%) with *Arabidopsis *AtDREB1D/CBF4 protein. MtCBF4 protein had a conserved AP2 DNA-binding domain and a C-terminal LWSY motif, as well as the CBF signature sequences (PKK/RPAGRxKFxETRHP, DSAWR, and A(A/V)xxA(A/V)xxF). However, the MtCBF4 protein contained DSAWK instead of DSAWR. These features are found in another CBF homolog, namely tobacco ACRE111B (GenBank accession no. AAG43549.1), which also contains DSAWK instead of DSAWR, indicating that the DSAWK might be a functional equivalent of DSAWR. MtCBF4 is a nuclear-localized protein and also possesses transactivation ability as expected.

Some transcription factors could be induced by a single stress, whereas others might be induced by multiple stresses. *AtCBF4 *was up-regulated by drought stress and ABA [21]. A previous study revealed that it was up-regulated by salt stress [18]. Moreover, transcription of *AtCBF4 *was induced under both chilling and cold stress, as indicated by the more sensitive qRT-PCR method [22]. Thus, *AtCBF4 *is induced under ABA, drought, cold, and salt stimulation. As another example, the transcription level of *GmDREB2 *was induced by cold, salt, and drought stress, as well as ABA treatment in soybeans [27]. *MtCBF4*, which exhibited a similar expression pattern, was also induced by ABA, drought, salt, and cold stimulation in our research. *MtCBF4 *might play an important role in response to abiotic stress in *M. truncatula*. Enriched presence of different stress-responsive *cis*-acting elements in the promoter of *MtCBF4 *explains why *MtCBF4 *was induced by multiple stresses and ABA treatment.

### *MtCBF4 *improved abiotic stress tolerance by activating downstream genes containing DRE elements

Researchers have shown that over-expression of DREB transcription factor genes improves stress tolerance [17,19,21,27,30,85,86]. Over-expression of *MtCBF4 *cDNA in transgenic *Arabidopsis *plants also activated some stress-inducible genes such as *COR15A, COR15B, KIN1, RD17, RD29A*, and *RD29B *under normal growing conditions. Among the downstream genes, *COR15A, COR15B, KIN1, RD29A *and *RD17*, which have A/GCCGACNT as the DRE core motif in their promoter regions, are *AtDREB1 *target genes [61,62] and showed a higher transcript level in *MtCBF4 *transgenic plants. By contrast, RD29B, which has ACCGACNA/G/C as the DRE core motif in its promoter region, belongs to the *AtDREB2A *specific regulon [63], and showed a comparatively lower expression level in the transgenic plants. As no probe sets were found significantly homologous to these downstream stress-inducible genes in *Arabidopsis*, we selected two other putative downstream genes of *MtCBF4*, namely *MtCAS15 *and *MtCAS31*, which also contain DRE elements in their promoter region, to check their expression changes in transgenic *M. truncatula*. Expression inducement of *MtCAS15 *and *MtCAS31 *was also detected in transgenic *M. truncatula *over-expressing *MtCBF4*. In our microarray data, expression of *MtCAS31 *was also up-regulated, but no homologous probe sets exist for *MtCAS15*. The results were consistent with the transactivation analysis in the yeast system. Collectively, these two analyses indicated MtCBF4 functions as a transcriptional activator.

## Conclusion

We used high-throughput microarray technology to monitor the expression dynamics in roots of *Medicago truncatula *seedlings in response to salt stress. Bioinformatic analysis of the expression data indicated that primary metabolism, including glycolysis, carbon fixation, starch, and sucrose metabolism, were affected most by external salt stress, whereas secondary metabolism pathways, which could help to reduce ROS threat and maintain osmotic balance, such as the anthocyanin and isoflavone pathways were induced, indicating an active response and defense mechanism protects the plant against external abiotic stress. In addition, we selected a transcription factor gene, *MtCBF4*, for function validation. Phylogenetic and multiple sequence alignment analysis revealed MtCBF4 is a novel member of the CBF transcriptional factor family in *M. truncatula*. The role in the abiotic stress response of *MtCBF4 *and the activation ability of downstream genes related to the stress response in transgenic *Arabidopsis *and *M. truncatula *over-expressing *MtCBF4 *indicated *MtCBF4 *is involved in stress tolerance, and is a good candidate gene for genetic improvement to produce stress-tolerant transgenic plants.

## Methods

### Plant material and treatments

Seeds of *Medicago truncatula *cv. Jemalong line A17 were obtained from the Biological Resource Centre for the model species *Medicago truncatula *(INRA BRC-MTR, http://www1.montpellier.inra.fr/BRC-MTR/accueil.php). Seeds were first soaked in concentrated sulfuric acid for about 25 min, then rinsed seven times with distilled H_2_O, incubated at 4°C for 2 d, and germinated on moistened filter papers. When the roots had grown to about 2 cm long, seeds were transplanted to a soil/vermiculite (1:1, v/v) mix and grown in a greenhouse maintained at 22°C under long-days (16 h light, 8 h dark) with 50% humidity. The plants were used for microarray experiments and expression pattern analyses of *MtCBF4 *under different stress assays.

Four weeks after germination, seedlings underwent several treatments. Treatment with ABA was performed by spraying 200 μM ABA solution containing 0.05% Tween 20 (v/v) on leaves for 1 h, 6 h and 24 h. For drought treatment, the seedlings were transferred to dry Whatman 3 MM paper in a sterile Petri dish for 1 h, 2 h and 3 h. For salt treatment, 180 mM NaCl for 6 h, 24 h and 48 h. For cold treatment, the seedlings were incubated at 4°C for 1 h, 6 h and 24 h. After treatment, the seedlings were harvested, frozen in liquid nitrogen immediately, and stored at -80°C for further analysis.

### Root length measurements under different NaCl concentrations

Young seedlings of *M. truncatula *were grown under different salt stress conditions (0, 60, 80, 90, 100, 110, 120, 140, 160, 180, or 200 mM NaCl) in darkness. Ten seedlings were treated with each NaCl concentration. Root lengths were measured at 12:00 on each day at 0, 1, 2, 3, 4, 5, 6, and 7 d after stress treatment was initiated. The percentage increase in root length per day was calculated as a measure of growth rate. The root lengths of seedlings treated with different NaCl levels were compared using Student's *t*-test.

### Microarray experiment, validation, and bioinformatic analysis

The design of the microarray experiment and data analysis was identical to that described in our previous work [34]. Original results of our micorarry experiments had also been deposited in NCBI GEO database [87] with accession number GSE13921 http://www.ncbi.nlm.nih.gov/geo/query/acc.cgi?acc=GSE13921. The same RNA from root samples was used for qRT-PCR analysis to confirm the microarray data.

Expression profiles were clustered using STEM software. Probe sets that were up- or down-regulated by more than two-fold at each time-point (compared to that at 0 h for each profile) were applied to function and pathway classification analysis using the GeneBins and PathExpress tools, respectively, using default parameters.

For phylogenetic analyses ClustalW version 1.83 [88] was used to generate the multiple alignment and MEGA version 4.1 [89] for phylogenetic reconstruction. Bootstrap support percentages were calculated from 1000 replications.

### *Arabidopsis *transgenic lines and treatments

*MtCBF4 *cDNA was amplified using reverse transcription PCR (RT-PCR) from mRNA extracted from four-week-old *M. truncatula *A17 plants treated with 200 mM NaCl for 6 h. The cDNA was cloned into the pMD18-T simple vector (TaKaRa, Dalian, China) using the primers 5'-TAC CAT GGA CAT GTT TAC TAT GAA TCA ATT-3' (*Nco *I site underlined) and 5'-ATA CTA GTT TAA AAT GAG TAA CTC CAC A-3' (*Spe *I site underlined). An *Nco*I-*Spe*I fragment containing *MtCBF4 *cDNA was inserted into pCAMBIA1302 containing the 35S CaMV promoter and a hygromycin (kanamycin) resistance marker. The plasmid was introduced into *Agrobacterium *EHA105 using heat shock. The pCAMBIA1302 vector containing *35S:MtCBF4 *was transformed into *Arabidopsis *plants using the floral dip method [90]. Genomic PCR, RT-PCR, and hygromycin spray/paint (80 mg ml^-1^) all confirmed the successful transfer of *35S:MtCBF4*.

To evaluate drought stress, one-week-old seedlings were transferred to pots (10 cm diameter) filled with a soil/vermiculite (1:1, v/v) mix for another 2 weeks, in a greenhouse under conditions of continuous illumination of approximately 100 μmol m^-2 ^s^-1^, 50% relative humidity, a temperature of 22°C, and long days (16 h light, 8 h dark), with regular watering every 4 d before water was withheld. After 16 d without water, all pots were watered simultaneously and plant recovery and survival rate were measured after 4 d.

To determine the sensitivity of seed germination to NaCl, seeds from wild-type and transgenic plants were placed on Murashige and Skoog (MS) agar plates [91] or MS agar plates saturated with 220 mM NaCl. The seeds were incubated at 4°C for 72 h before transfer to a growth chamber with constant light (approximately 100 μmol m^-2 ^s^-1^) at 22°C for germination. After 7 d, seed germination was recorded. Seeds were considered to have germinated when radicles were 1 mm long.

To assess salt tolerance, three-day old seedlings were carefully transferred to MS medium containing different concentrations of NaCl (0, 50, 100, 125, 150, or 175 mM) for 7 d. Seedling root lengths were analyzed with Image software http://rsb.info.nih.gov/ij/index.html. The drought and salt tolerance experiments were repeated three times.

### qRT-PCR analysis

Total RNA was extracted from plants harvested at the specified time-points with TRIzol reagent (Invitrogen, USA) and treated with RNase-free DNaseI (Progema). Total RNA (2 μg) was used for reverse transcription with M-MLV Reverse Transcriptase (Promega) and the cDNA samples were diluted by four-fold. For qRT-PCR, triplicate quantitative assays were performed on each cDNA dilution with SYBR Premix Ex Taq (TaKaRa) and a CFX Manager sequence detection system according to the following protocol: denaturation at 95°C with 30 s for initiation, denaturation at 95°C for 10 s, 40 cycles of amplification, annealing and extension at 51°C/57.6°C for 30 s, and collection of fluorescence data at 51°C by reading the plates. Specificity of the amplification was checked using a melting curve performed from 65-95°C, as well as sequencing of the amplicon. Three independent replicates were performed per experiment, and the means and corresponding standard errors were calculated.

For *MtCBF4 *expression pattern analysis as well as transient expression assays in *M. truncatula*, the annealing and extension temperature is 51°C; *MtActin *gene was used in parallel to perform the reaction and to control constitutive expression.

For detection of the transcript levels of target genes, the annealing and extension temperature was 57.6°C. *AtACTIN2 *and *Atβ-TUBULIN *were used in parallel to perform the reaction and to control constitutive expression. All relevant primer sequences used in this work are listed in Additional file 3.

### Localization of MtCBF4-GFP fusion proteins

The entire coding sequence of *MtCBF4 *was amplified with two primers: 5'-CTC GAG GAT GTT TAC TAT GAA TCA ATT TTC-3' (*Xho*I site underlined) and 5'-GGT ACC CAA ATG AGT AAC TCC ACA ATG AAA CT-3' (*Kpn*I site underlined). The PCR product was subcloned into the pE3025-GFP vector to generate p*E3025-MtCBF4-GFP *containing an *MtCBF4-GFP *fusion construct under the control of the CaMV dual 35S promoter, as well as a TEV enhancer. The construct was confirmed by sequencing and used for transient transformation of onion (*Allium cepa*) epidermal cells via a gene gun (Bio-Rad, California, USA). GFP fluorescence was observed under a confocal microscope (Nikon). The pE3025-GFP empty vector was used as a control.

In this study, the pE3025-GFP vector was derived from the pSATS-RFP-NI vector. Using *Xma*I and *Xba*I restriction enzymes, we replaced the red fluorescent protein (RFP) gene with the *GFP *gene, which was cloned from pCAMBIA1302 with the primers 5'-ATC CCG GGA TGG TAG ATC TGA CTA GT-3' (*Xma*I site underlined) and 5'-ATTCT AGA TTA GTG GCT AGC T'TT GTA TAG-3' (*Xba*I site underlined). This created a new vector named pE3025-GFP.

### Transactivation analysis in yeast

The entire coding sequence of *MtCBF4 *was amplified using two primers: 5'-GAA TTC ATG TTT ACT ATG AAT CAA TTT TC-3' (*EcoR*I site underlined) and 5'-CTG CAG TTA AAA TGA GTA ACT CCA CAA TG-3' (*Pst*I site underlined). The PCR product was subcloned into the DNA-binding domain vector pBD GAL4, a yeast expression vector with the promoter and terminator of the *ADH1 *gene, to construct GAL4 DNA-BD-MtCBF4 fusion plasmids pBD-MtCBF4. The recombinant plasmid was then transferred into a yeast strain YRG-2 carrying the reporter genes *His3 *and *LacZ*. The yeast strain cannot grow on the SD plates without histidine, which cannot induce LacZ (β-galactosidase) activity. The transformed yeast culture was dropped onto SD plates without tryptophan or without both tryptophan and histidine. The plates were incubated at 30°C for 3 d and applied to a β-Gal assay to examine the transactivation ability of MtCBF4.

### Transient expression assays and preparation of *A. rhizogenes*-transformed roots

*Agrobacterium rhizogenes*-transformed *M. truncatula *roots were prepared as described previously [64]. Three weeks after inoculation of seedling roots without apices with *A. rhizogenes *strain Arqua1, the seedlings were transferred to a new plate with Fahraeus medium containing 100 mM NaCl. At the moment of transfer, the position of the root apex was labeled and root length was measured 1 week after transfer. Three biological experiments were performed, and at least 100 independent transgenic roots per construct and per condition were analyzed. For over-expression of *MtCBF4*, an *Nco *I-*Spe *I fragment containing *MtCBF4 *cDNA was inserted into the pRNAi vector containing the d35S promoter and an OCS 3', and amplified with the same primers used to construct pCAMBIA1302-MtCBF4. The resulting inverted construct was inserted *Kpn *I-*Pac *I into the pRedRoot binary vector [64]. The transgenic lines were determined by detecting RFP.

For transient expression assays in *M. truncatula, A. rhizogenes *strain Arqua1 carrying the same constructs was used for vacuum infiltration of four-week-old *M. truncatula *A17 plants as described previously [32]. After transfection, plants were grown in a growth chamber at 24°C under long days (16 h light, 8 h dark) for 48 h. For salt treatment, the leaves were treated with 100 mM NaCl for 6 h, and ddH_2_O treatment was used as a control. Leaves were then collected for total RNA extraction to perform qRT-PCR analysis. Expression of *MtCAS15, MtCAS31 *and *MtCBF4 *was normalized using *MtActin*.

## Authors' contributions

DL, YZ and JD wrote the manuscript. DL performed the microarray experiment and bioinformatics data analysis, YZ performed *MtCBF4*-related experiments, XH, XS and LM provided assistance. ZS, TW and JD designed and supervised this work. All authors read and approved the final manuscript.

## Supplementary Material

Additional file 1 **Root length data for *Medicago truncatula *seedlings grown under salt stress**. Ten biological replicates for each NaCl concentration. The unit used is centimeter. The calculations and statistical test results are also available in this file. Click here for file

Additional file 2 **qRT-PCR validation of microarray results**. Fold changes in expression of five probe sets obtained from qRT-PCR and microarray experiments. Data represent the fold change in expression level at the respective time-point relative to that at 0 h. Error bars indicate SE. The primers used are listed in Additional file 3 (Table S1). Click here for file

Additional file 3 **Primers used for qRT-PCR**. The primers used for *MtActin *were validated by the utility In Silico PCR of the MtED database http://bioinformatics.cau.edu.cn/MtED/blast/cgi-bin/webPcr. The *Arabidopsis *Genome Initiative (TAIR) locus identifiers for the genes mentioned in this article are as follows: *RD29A *(At5g52310), *RD29B *(At5g52300), *RD17 *(At1g20440), *COR15A *(At2g42540), *COR15B *(At2g42530), *KIN1 *(At5g15960), *ACTIN*2 (AT3G18780), *β-TUBULIN *(At5g12250). *Medicago truncatula *Genome Initiative locus identifiers for the genes mentioned in this article are as follows: *MtCAS15 *(EU139869.1), *MtCAS31 *(EU139871.1). Primers for *MtCBF4 *and *MtActin *in the MtCBF4 expression pattern and transient transfection experiments were the same as those used in the qRT-PCR validation of the microarray experiment. Click here for file

Additional file 4 **Summary of GeneBins analysis**. The number in the first line of a cell indicates the number of probes assigned to this GeneBins ontology. Lower-case letters in the second line indicate the STEM profile identification, which we named statistically significant STEM profiles from *a *to *k*. The number following the colon indicates the number of probes assigned to the corresponding STEM profile. Each STEM profile and the corresponding number of probes are separated by a semicolon. Click here for file

Additional file 5 **Cluster analysis results of 2138 transcription factors**. The 2138 transcription factors were chosen for re-cluster analysis. The red star indicates the profile that *MtCBF4 *belongs to. Click here for file

Additional file 6 **Phylogenetic analysis of DREB/CBF family proteins**. Proteins from different species are indicated by different colors. DREB/CBFs from *Arabidopsis *are shown in green: AtDREB1B/CBF1 (NP_567721.1), AtDREB1C/CBF2 (NP_567719.1), AtDREB1A/CBF3 (NP_567720.1), AtDREB1D/CBF4 (NP_200012.1), AtDREB2C (Q8LFR2.2), AtDDF1 (NP_172721.1), AtDDF2 (NP_176491.1), AtDREB2A (NP_001031837.1), and AtDREB2B (NP_187713.1). DREB/CBFs from *Medicago *are shown in red: MtCBF1 (ABX80062.1), MtCBF2 (ABX80063.1), MtDREB1C/CBF3 (ABX80064.1), and MtCBF4 (ADL74429.1). DREB/CBFs from soybean are shown in blue: GmCBF1 (ACA64423.1), GmCBF2 (ACB45077.1), GmCBF3 (ACA63936.1), GmDERBa (AAT12423.1), GmDREBb (AAQ57226.1), GmDREBc (AAP83131.1), GmDREB1 (AF514908.1), GmDREB2 (ABB36645.1), and GmDREB3 (AAZ03388.1). The phylogenetic tree was constructed using Mega software with neighbor-joining method. The numbers shown beside the branches are bootstrap probabilities from 1000 replications. Click here for file

Additional file 7 **Promoter sequence analysis of *MtCBF4***. The 1000 bp upstream from the translation start site of *MtCBF4 *was scanned by the PLACE tool for transcriptional factor binding-site analysis. Sites are listed according to their position at the promoter. Click here for file

Additional file 8 **Effect of over-expression of *MtCBF4 *on plant growth under normal conditions**. (a) Inflorescence heights of eight-week-old wild-type plants and *35S:MtCBF4 *plants. Average inflorescence heights were calculated from 15 plants. Error bars show the SD. No significant difference was detected between *MtCBF4 *transgenic lines and WT plants. (b) Seeds were harvested from three-month-old wild-type plants and *35S:MtCBF4 *plants and air-dry seeds were weighed. The average yield of each line was calculated from yields of 15 plants. Error bars show the SD. No significant difference was detected between *MtCBF4 *transgenic lines and WT plants. Click here for file

Additional file 9 **Expression of *MtCBF4 *improved salt tolerance in *M. truncatula***. Another two representative cultivars of *MtCBF4*-overexpressing *A. rhizogenes*-transformed *M. truncatula *roots 1 week after transfered to control medium (left) and medium containing 100 mM NaCl (right). Click here for file
