# Supplementary figures and images for: Transcriptional profiling of Medicago truncatula under salt stress identified a novel CBF transcription factor MtCBF4 that plays an important role in abiotic stress responses
Source: BMC Plant Biol. 2011 Jul 1;11:109. doi: 10.1186/1471-2229-11-109 (PMC3146422; doi:10.1186/1471-2229-11-109)

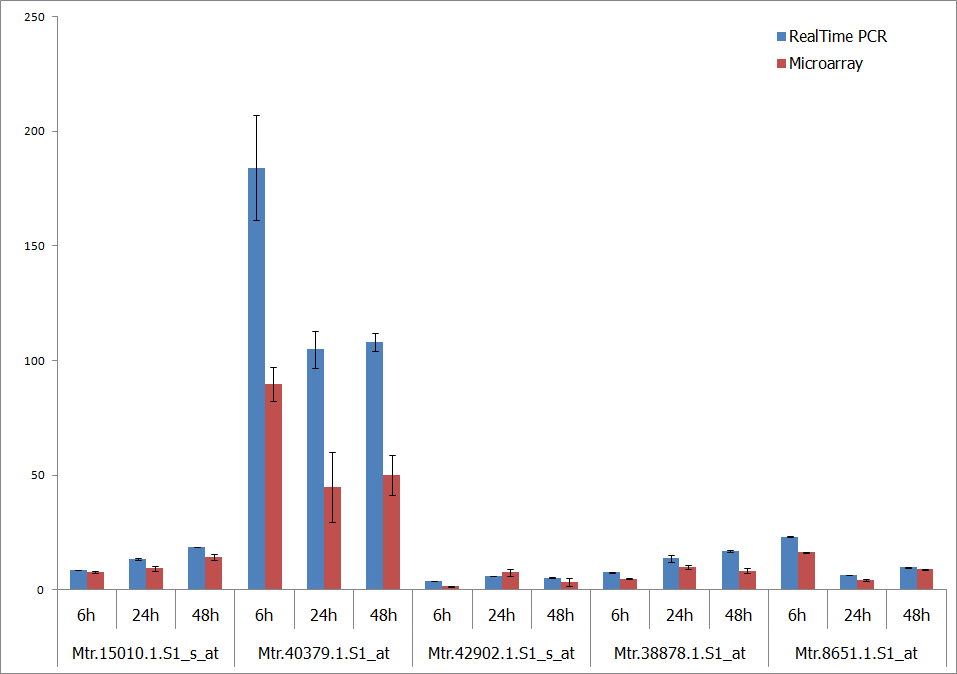

Supplement: Additional file 2 — qRT-PCR validation of microarray results. Fold changes in expression of five probe sets obtained from qRT-PCR and microarray experiments. Data represent the fold change in expression level at the respective time-point relative to that at 0 h. Error bars indicate SE. The primers used are listed in Additional file 3 (Table S1). [file 1471-2229-11-109-S2.TIFF]

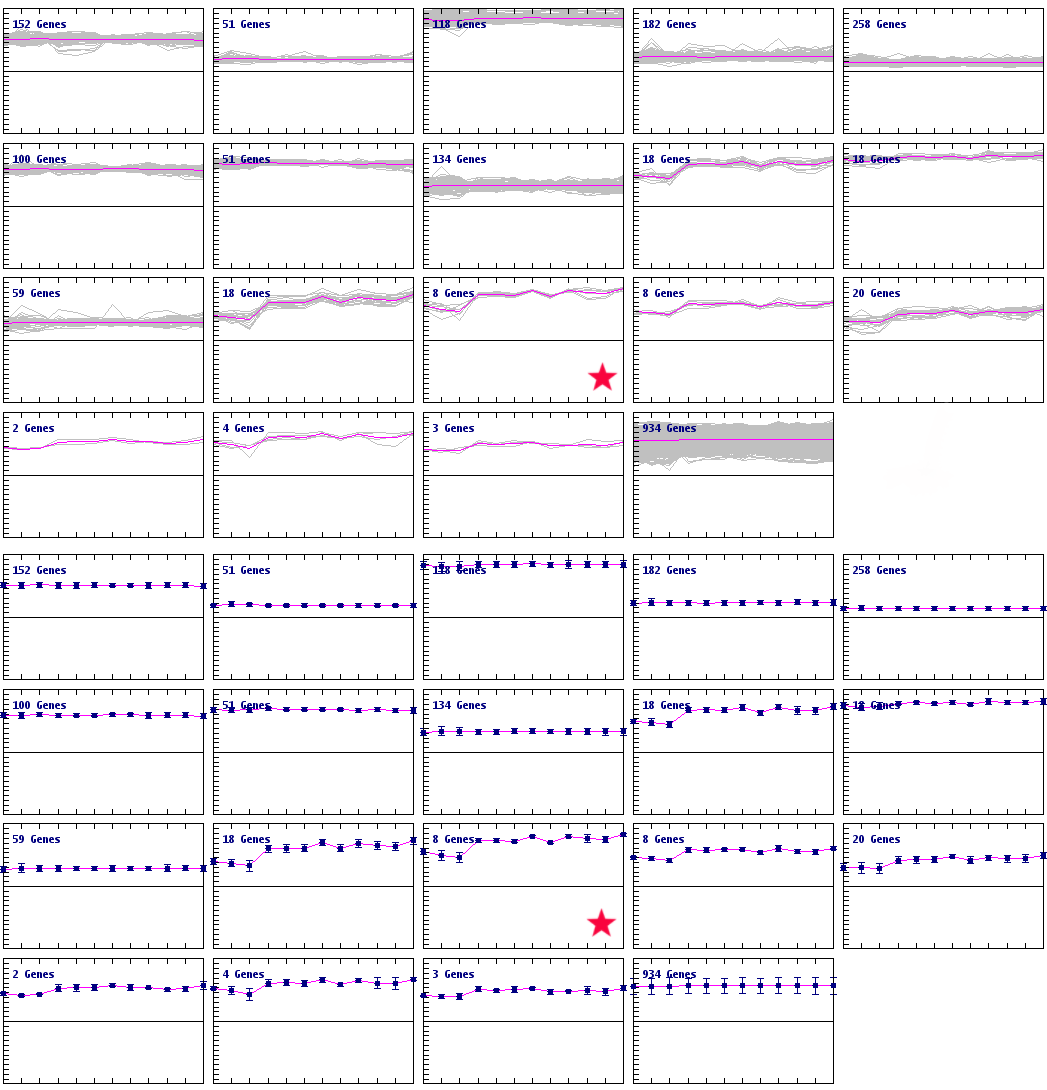

Supplement: Additional file 5 — Cluster analysis results of 2138 transcription factors. The 2138 transcription factors were chosen for re-cluster analysis. The red star indicates the profile that MtCBF4 belongs to. [file 1471-2229-11-109-S5.TIFF]

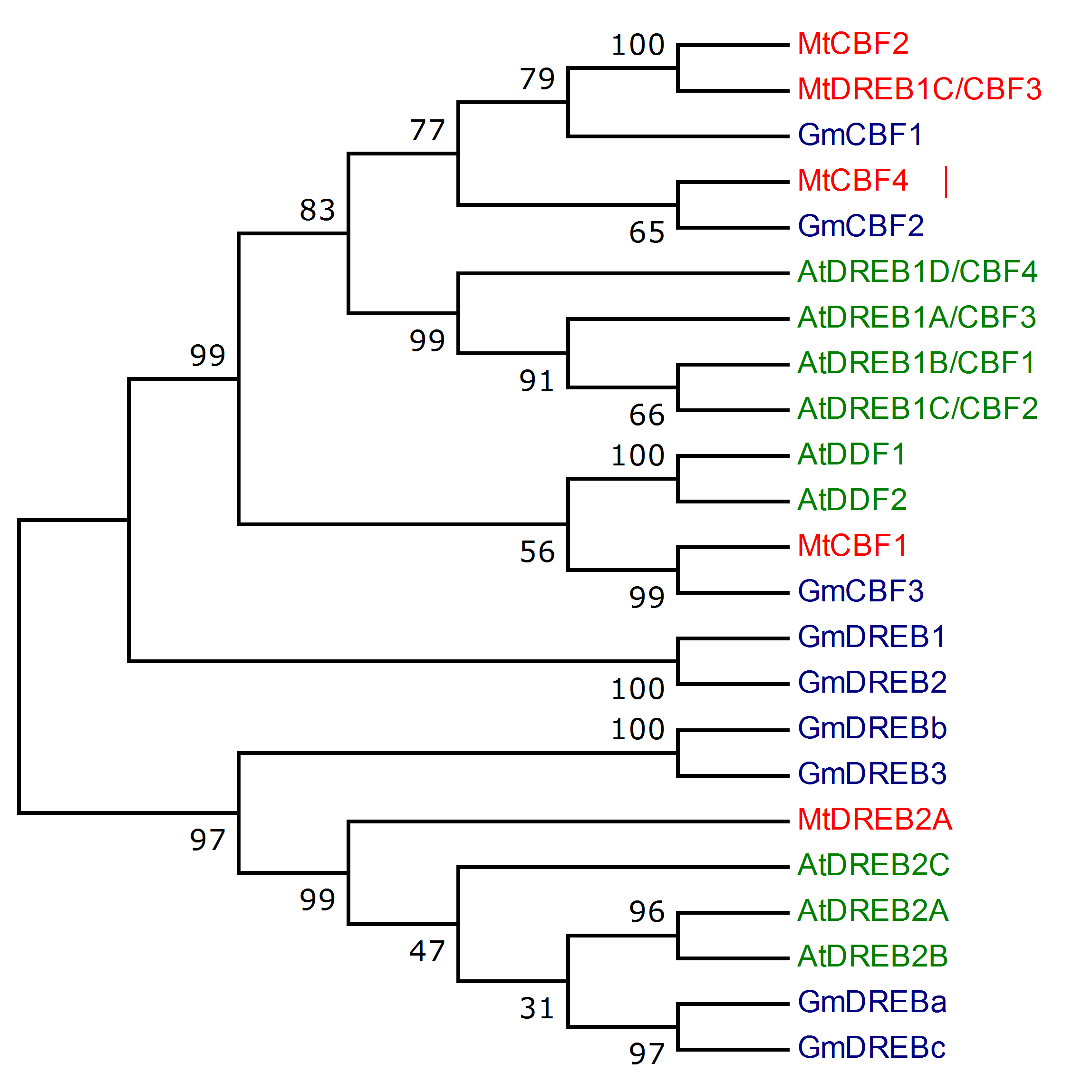

Supplement: Additional file 6 — Phylogenetic analysis of DREB/CBF family proteins. Proteins from different species are indicated by different colors. DREB/CBFs from Arabidopsis are shown in green: AtDREB1B/CBF1 (NP_567721.1), AtDREB1C/CBF2 (NP_567719.1), AtDREB1A/CBF3 (NP_567720.1), AtDREB1D/CBF4 (NP_200012.1), AtDREB2C (Q8LFR2.2), AtDDF1 (NP_172721.1), AtDDF2 (NP_176491.1), AtDREB2A (NP_001031837.1), and AtDREB2B (NP_187713.1). DREB/CBFs from Medicago are shown in red: MtCBF1 (ABX80062.1), MtCBF2 (ABX80063.1), MtDREB1C/CBF3 (ABX80064.1), and MtCBF4 (ADL74429.1). DREB/CBFs from soybean are shown in blue: GmCBF1 (ACA64423.1), GmCBF2 (ACB45077.1), GmCBF3 (ACA63936.1), GmDERBa (AAT12423.1), GmDREBb (AAQ57226.1), GmDREBc (AAP83131.1), GmDREB1 (AF514908.1), GmDREB2 (ABB36645.1), and GmDREB3 (AAZ03388.1). The phylogenetic tree was constructed using Mega software with neighbor-joining method. The numbers shown beside the branches are bootstrap probabilities from 1000 replications. [file 1471-2229-11-109-S6.TIFF]

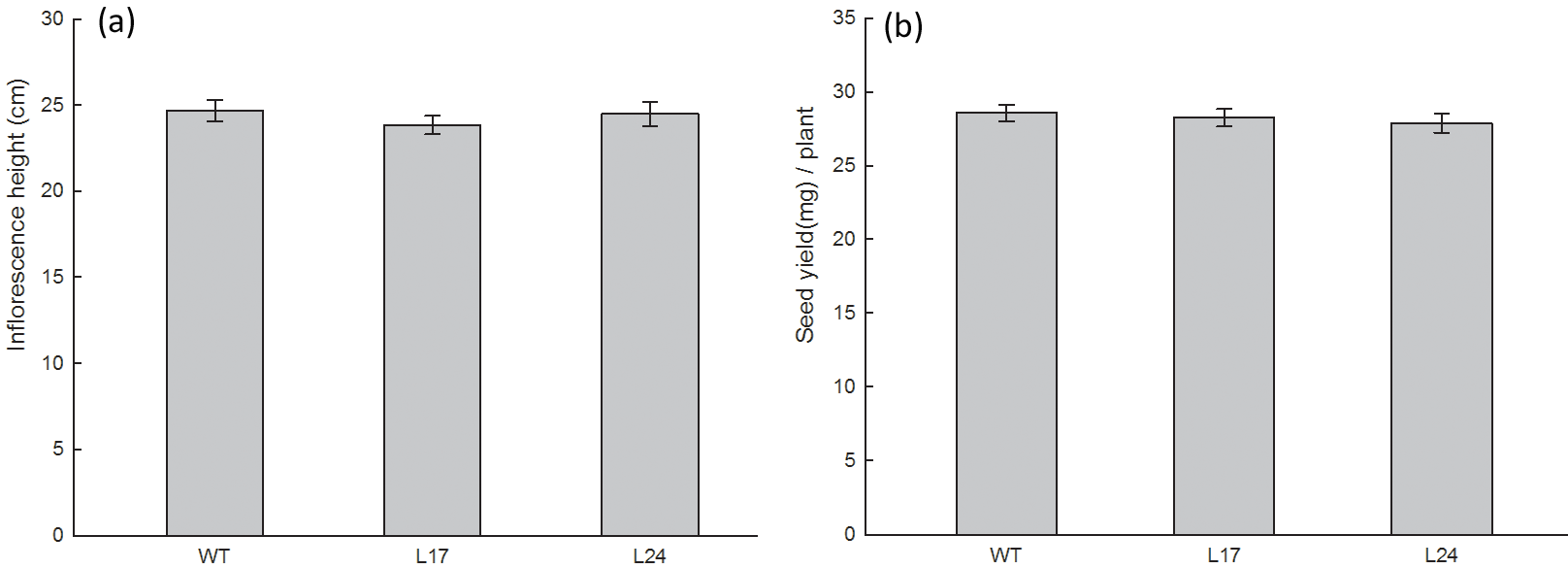

Supplement: Additional file 8 — Effect of over-expression of MtCBF4 on plant growth under normal conditions. (a) Inflorescence heights of eight-week-old wild-type plants and 35S:MtCBF4 plants. Average inflorescence heights were calculated from 15 plants. Error bars show the SD. No significant difference was detected between MtCBF4 transgenic lines and WT plants. (b) Seeds were harvested from three-month-old wild-type plants and 35S:MtCBF4 plants and air-dry seeds were weighed. The average yield of each line was calculated from yields of 15 plants. Error bars show the SD. No significant difference was detected between MtCBF4 transgenic lines and WT plants. [file 1471-2229-11-109-S8.TIFF]

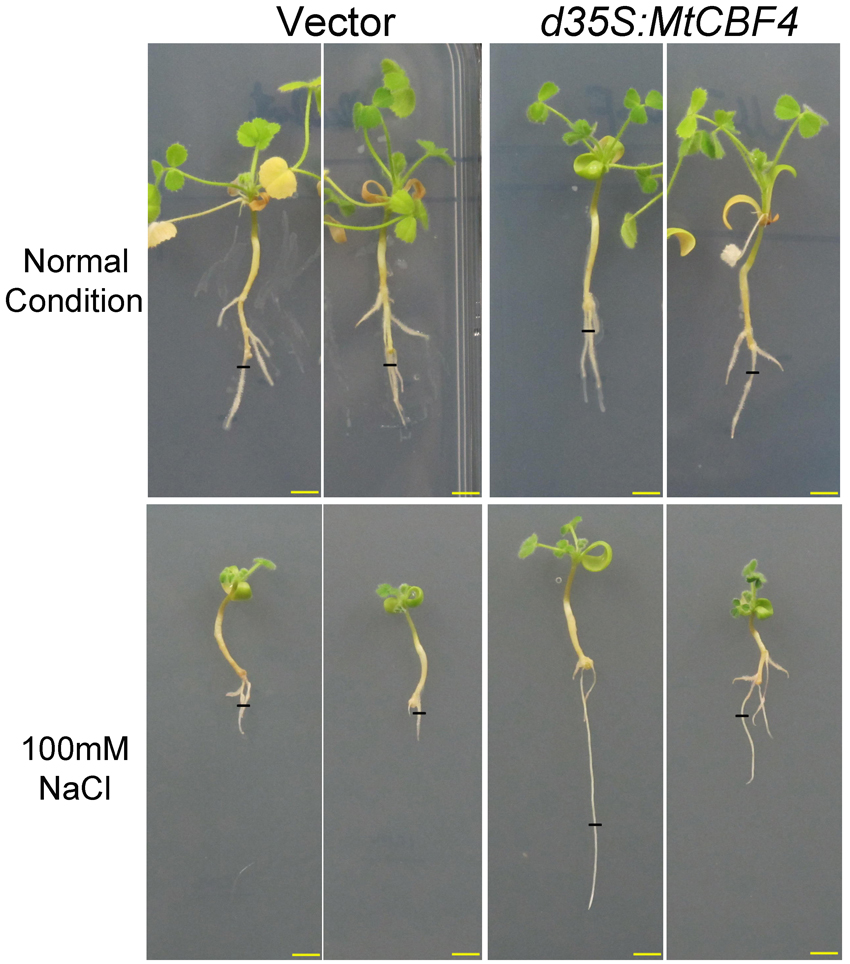

Supplement: Additional file 9 — Expression of MtCBF4 improved salt tolerance in M. truncatula. Another two representative cultivars of MtCBF4-overexpressing A. rhizogenes-transformed M. truncatula roots 1 week after transfered to control medium (left) and medium containing 100 mM NaCl (right). [file 1471-2229-11-109-S9.JPEG]
