# Supplementary material for: Transcriptional profiling of Medicago truncatula under salt stress identified a novel CBF transcription factor MtCBF4 that plays an important role in abiotic stress responses
Source: BMC Plant Biol. 2011 Jul 1;11:109. doi: 10.1186/1471-2229-11-109 (PMC3146422; doi:10.1186/1471-2229-11-109)
Supplement: Additional file 3 — Primers used for qRT-PCR. The primers used for MtActin were validated by the utility In Silico PCR of the MtED database http://bioinformatics.cau.edu.cn/MtED/blast/cgi-bin/webPcr. The Arabidopsis Genome Initiative (TAIR) locus identifiers for the genes mentioned in this article are as follows: RD29A (At5g52310), RD29B (At5g52300), RD17 (At1g20440), COR15A (At2g42540), COR15B (At2g42530), KIN1 (At5g15960), ACTIN2 (AT3G18780), β-TUBULIN (At5g12250). Medicago truncatula Genome Initiative locus identifiers for the genes mentioned in this article are as follows: MtCAS15 (EU139869.1), MtCAS31 (EU139871.1). Primers for MtCBF4 and MtActin in the MtCBF4 expression pattern and transient transfection experiments were the same as those used in the qRT-PCR validation of the microarray experiment. [file 1471-2229-11-109-S3.PDF]

Primers used for Quantitative Real-time PCR in this article are listed below.

Table S1 Primers used for Quantitative real-time PCR validation of microarray experiments are listed below.

Probe Set	Primer Sequence 5'→3'		Amplification Size(bp)
Mtr.40379.1.S1_at	Forward Primer	CAGCAATGACCTATGATGAATACA	186
	Reverse Primer	AACCTACCCAACCCAAGAGC	
Mtr.42902.1.S1_s_at	Forward Primer	CAATGACAGTTTGGCTGGTCT	111
	Reverse Primer	TTTGAGTTTGGGTCACTTGGA	
Mtr.8651.1.S1_at	Forward Primer	AACAAAATACTTATGGGACAGGC	318
	Reverse Primer	GTTACATACGAACCAACTCACTCA	
Mtr.38878.1.S1_at	Forward Primer	CGGAAGCAAGGGATATTCAA	246
	Reverse Primer	CAAATTCCACGTCAGCAACA	
Mtr.15010.1.S1_s_at	Forward Primer	TCCCCGACACGAATAATCCA	233
	Reverse Primer	ACAAGCACCGCTCTTCTCCT	
MtActin	Forward Primer	CCCACTGGATGTCTGTAGGTT	197
	Reverse Primer	AGAATTAAGTAGCAGCGCAAA	

The primers used for *MtActin* could be validated by the utility In Silico PCR of MtED database (http://bioinformatics.cau.edu.cn/MtED/blast/cgi-bin/webPcr).

Table S2 Primers used for Quantitative real-time PCR expression analysis of genes downstream of *MtCBF4* in *Arabidopsis*.

Gene	Primer Sequence 5'→3'		Amplification Size(bp)
RD29A	Forward primer	ATC ACT TGG CTC CAC TGT TGT TC	98
	Reverse primer	ACA AAA CAC ACA TAA ACA TCC AAA GT	
RD29B	Forward primer	GAA TCA AAA GCT GGG ATG GA	196
	Reverse primer	TGCTCTGTGTAGGTGCTTGG	
COR15A	Forward primer	CTC AGT TCG TCG TCG TTT C	147
	Reverse primer	CAT CTGCTA ATG CCT CTT T	
COR15B	Forward primer	GGCGATGTCTTTATCAGGAG	127
	Reverse primer	CTGAGCAACGACGACGAG	
RD17	Forward primer	TAT CAT GCC AAG ACCACT GAA	157
	Reverse primer	CAA CGA AAG CCA CAA TAA CAA	
KIN1	Forward primer	ACC AAC AAG AAT GCC TTC CA	147
	Reverse primer	CCG CAT CCG ATA CAC TCT TT	
ACTIN2	Forward primer	GGTAAC ATT GTG CTC AGT GGT GG	108
	Reverse primer	AAC GAC CTTAAT CTT CATGCT GC	
β-TUBULIN	Forward primer	TGG GAA CTC TGC TCA TAT CT	340

	Reverse primer	GAA AGG AAT GAG GTT CAC TG

Arabidopsis Genome Initiative locus identifiers for the genes mentioned in this article are as follows: *RD29A* (At5g52310), *RD29B* (At5g52300), *RD17* (At1g20440), *COR15A* (At2g42540), *COR15B* (At2g42530), *KIN1* (At5g15960), *ACTIN*2 (AT3G18780), *β-TUBULIN* (At5g12250).

Table S3 Primers used for Quantitative real-time PCR expression analysis of genes downstream of *MtCBF4* in *Medicago truncatula*.

Gene	Primer Sequence 5'→3'		Amplification Size(bp)
MtCAS15	Forward primer	GAG AAC ACC ACG GTG AGT ACA A	237
	Reverse primer	CAT GTT CAT GAC CCT CTC CA	
MtCAS31	Forward primer	CAT GGA CAT CAT CAA CAA CAT C	200
	Reverse primer	CCA CCA ATG TCA GTG TTT CC	

Medicago truncatula Genome Initiative locus identifiers for the genes mentioned in this article are as follows: *MtCAS15* (EU139869.1), *MtCAS31* (EU139871.1). Primers of *MtCBF4* and *MtActin* were the same as that in the experiment of real-time PCR validation of microarray.
