# Supplementary material for: Transcriptional profiling of Medicago truncatula under salt stress identified a novel CBF transcription factor MtCBF4 that plays an important role in abiotic stress responses
Source: BMC Plant Biol. 2011 Jul 1;11:109. doi: 10.1186/1471-2229-11-109 (PMC3146422; doi:10.1186/1471-2229-11-109)
Supplement: Additional file 4 — Summary of GeneBins analysis. The number in the first line of a cell indicates the number of probes assigned to this GeneBins ontology. Lower-case letters in the second line indicate the STEM profile identification, which we named statistically significant STEM profiles from a to k. The number following the colon indicates the number of probes assigned to the corresponding STEM profile. Each STEM profile and the corresponding number of probes are separated by a semicolon. [file 1471-2229-11-109-S4.PDF]

Summary of GeneBins analysis.

Pathway classification	Up-regulated			Down-regulated		
	6h	24h	48h	6h	24h	48h
**Metabolism**						
Carbohydrate Metabolism	296 b:24;d:112;e:43;f:2;h:1;k:29	448 b:78;d:165;e:48;f:58;k:33	475 b:114;d:127;e:60;f:64;k:23	294 a:54;c:102;g:63;i:12;j:22	472 a:136;c:105;g:63;h:54;i:19;j:43	637 a:185;c:138;g:67;h:58;i:60;j:29
Energy Metabolism	83 b:4;d:30;e:12;f:1;k:9	114 b:24;d:36;e:10;f:18;k:9	119 b:32;d:31;e:18;f:19;k:3	127 a:28;c:36;g:35;i:1;j:2	188 a:64;c:35;g:32;h:25;i:2;j:9	221 a:81;c:46;g:34;h:24;i:8;j:3
Lipid Metabolism	160 b:14;d:39;e:23;f:2;h:1;k:19	204 b:49;d:52;e:24;f:21;k:18	226 b:65;d:42;e:30;f:24;k:13	136 a:35;c:36;g:25;h:1;i:2;j:8	244 a:89;c:42;g:23;h:28;i:8;j:16	302 a:106;c:51;g:26;h:33;i:23;j:9
Nucleotide Metabolism	32 b:5;d:12;e:4;k:2	52 b:15;d:18;e:4;f:5;k:2	52 b:20;d:14;e:8;f:4;k:1	58 a:2;c:30;g:12;j:1	86 a:18;c:31;g:14;h:8;i:1;j:5	126 a:30;c:51;g:12;h:9;i:10;j:3
Amino Acid Metabolism	197 b:12;d:48;e:39;f:6;h:1;k:19	234 b:53;d:58;e:37;f:28;k:19	274 b:79;d:50;e:53;f:28;k:14	148 a:34;c:43;g:34;j:9	265 a:89;c:53;g:35;h:32;i:4;j:25	319 a:109;c:74;g:31;h:36;i:15;j:14
Metabolism of Other Amino Acids	99 b:3;d:28;e:18;f:2;k:12	110 b:16;d:35;e:18;f:13;k:11	111 b:26;d:28;e:27;f:13;k:6	64 a:8;c:21;g:10;j:8	116 a:40;c:20;g:12;h:8;i:1;j:16	133 a:48;c:30;g:11;h:11;i:2;j:10
Glycan Biosynthesis and Metabolism	150 b:12;d:64;e:24;f:3;k:15	244 b:34;d:101;e:27;f:37;k:19	241 b:49;d:78;e:31;f:38;k:11	113 a:19;c:52;g:20;i:2;j:3	196 a:62;c:54;g:22;h:22;i:9;j:10	273 a:82;c:72;g:25;h:24;i:27;j:3
Biosynthesis of Polyketides and Nonribosomal Peptides	7 d:1;e:2;k:1	8 b:1;d:1;e:2;k:1	10 b:2;d:1;e:2;f:1;k:1	9 a:3;c:4;g:1	19 a:9;c:6;g:1;h:3	28 a:9;c:8;g:1;h:4;i:1
Metabolism of Cofactors and Vitamins	179 b:15;d:76;e:13;f:2;k:21	289 b:45;d:117;e:19;f:34;k:26	262 b:57;d:89;e:25;f:32;k:13	105 a:18;c:44;g:24;i:2;j:4	183 a:59;c:44;g:29;h:22;i:6;j:10	269 a:83;c:69;g:31;h:21;i:28;j:6
Biosynthesis of Secondary Metabolites	231 b:20;d:59;e:55;f:4;h:1;k:29	291 b:68;d:65;e:57;f:30;k:27	324 b:84;d:57;e:67;f:31;k:19	178 a:37;c:42;g:49;h:1;i:1;j:20	316 a:84;c:47;g:50;h:56;i:7;j:35	342 a:97;c:53;g:49;h:63;i:16;j:26

Biodegradation of Xenobiotics	235<br>b:15;d:88;e:34;f:3;h:1;k:23	327<br>b:54;d:134;e:37;f:30;k:28	324<br>b:76;d:101;e:47;f:29;k:18	112<br>a:22;c:35;g:31;i:2;j:4	186<br>a:57;c:36;g:32;h:19;i:7;j:14	247<br>a:76;c:50;g:33;h:22;i:25;j:7
**Genetic Information Processing**						
Transcription	2<br>e:1	5<br>e:3;f:1	5<br>b:1;e:3;f:1	9<br>g:2	8<br>a:3;g:3;j:1	15<br>a:8;g:3;i:2
Translation	24<br>b:4;d:5;e:5;f:4;k:1	31<br>b:7;d:6;e:4;f:10;k:1	49<br>b:12;d:6;e:6;f:11	51<br>a:10;c:14;g:16;j:1	160<br>a:74;c:24;g:19;h:12;i:1;j:19	202<br>a:103;c:41;g:15;h:17;i:3;j:11
Folding Sorting and Degradation	83<br>b:4;d:22;e:26;f:1;k:6	100<br>b:9;d:34;e:26;f:18;k:7	120<br>b:17;d:27;e:37;f:22;k:5	63<br>a:2;c:27;g:20;j:3	122<br>a:26;c:34;g:25;h:8;j:19	154<br>a:41;c:53;g:22;h:8;i:13;j:7
Replication and Repair	11<br>b:2;d:5;e:1;k:1	30<br>b:6;d:14;e:1;f:4;k:1	34<br>b:8;d:11;e:3;f:5;k:1	21<br>a:2;c:11;g:6;i:1	44<br>a:11;c:12;g:8;h:3;i:1;j:5	60<br>a:13;c:19;g:8;h:4;i:7;j:3
**Environmental Information Processing**						
Membrane Transport	87<br>b:8;d:27;e:15;k:11	119<br>b:19;d:37;e:15;f:17;k:12	134<br>b:28;d:32;e:23;f:19;k:7	47<br>a:8;c:14;g:9;i:4;j:1	88<br>a:22;c:17;g:12;h:17;i:5;j:3	111<br>a:29;c:26;g:10;h:19;i:10;j:1
Signal Transduction	280<br>b:21;d:120;e:44;f:6;k:33	454<br>b:56;d:188;e:52;f:68;k:36	432<br>b:82;d:145;e:68;f:69;k:20	115<br>a:15;c:54;g:24;i:5;j:1	198<br>a:60;c:59;g:25;h:12;i:7;j:16	282<br>a:93;c:85;g:29;h:13;i:24;j:7
Ligand-Receptor Interaction	147<br>b:11;d:80;e:17;f:4;k:16	271<br>b:31;d:121;e:25;f:48;k:19	246<br>b:44;d:99;e:27;f:47;k:8	55<br>a:9;c:19;g:17;i:4;j:1	79<br>a:21;c:15;g:16;h:7;i:5;j:9	119<br>a:30;c:28;g:19;h:7;i:18;j:2
**Cellular Processes**						
Cell Motility	28<br>b:1;d:9;e:7;k:4	45<br>b:7;d:17;e:6;f:7;k:3	50<br>b:11;d:14;e:11;f:9;k:2	18<br>a:4;c:5;g:5;j:1	26<br>a:8;c:5;g:3;h:1;j:5	39<br>a:10;c:11;g:5;h:1;i:3;j:2
Cell Growth and Death	176<br>b:14;d:92;e:24;f:2;k:20	330<br>b:36;d:146;e:29;f:51;k:27	296<br>b:60;d:107;e:37;f:51;k:9	99<br>a:11;c:56;g:15;i:7;j:2	146<br>a:32;c:58;g:17;h:10;i:7;j:11	213<br>a:51;c:78;g:23;h:9;i:25;j:3
Cell Communication	29<br>b:6;d:8;e:7;k:2	52<br>b:12;d:18;e:8;f:8;k:2	55<br>b:18;d:13;e:12;f:8;k:1	22<br>a:4;c:11;g:1;i:3;j:1	44<br>a:15;c:12;g:1;h:4;i:3;j:	59<br>a:18;c:21;g:1;h:3;i:6;j:

					3	1
Immune System	2<br>d:1	1<br>d:1	1<br>d:1	0	0	0
Development	17<br>b:1;d:4;e:5;k:2	32<br>b:6;d:11;e:4;f:6;k:2	34<br>b:9;d:8;e:8;f:6;k:1	10<br>c:6;g:2;i:1	20<br>a:6;c:6;g:2;h:1;i:1;j:1	27<br>a:9;c:10;g:2;h:1;i:2
Behavior	1<br>b:1	3<br>b:1;d:1;f:1	4<br>b:2;d:1;f:1	0	0	0
**Unclassified**						
Unclassified with homolog	1153<br>b:80;d:376;e:209;f:9;k:119	1516<br>b:288;d:505;e:233;f:150;k:119	1654<br>b:430;d:425;e:314;f:157;k:67	958<br>a:156;c:290;g:227;h:7;i:25;j:50	1653<br>a:496;c:317;g:243;h:191;i:45;j:158	2160<br>a:679;c:467;g:240;h:207;i:166;j:94
Unclassified without homolog	582<br>b:51;d:194;e:117;f:14;k:61	1094<br>b:235;d:302;e:144;f:191;k:57	1283<br>b:409;d:224;e:204;f:198;k:30	387<br>a:51;c:108;g:112;h:1;i:9;j:32	696<br>a:190;c:138;g:122;h:64;i:9;j:99	859<br>a:257;c:202;g:125;h:64;i:69;j:44

Number in the first line of a cell indicates number of probes assigned to this GeneBins ontology, lowercase letters in the second line indicates the STEM profile id, which we named statistic significant STEM profiles from *a* to *k*, the number behind the colon means number of probes assigned to corresponding STEM profile, each STEM profile and the corresponding number of probes were separated by semicolon.
