# Supplementary material for: Transcriptional profiling of Medicago truncatula under salt stress identified a novel CBF transcription factor MtCBF4 that plays an important role in abiotic stress responses
Source: BMC Plant Biol. 2011 Jul 1;11:109. doi: 10.1186/1471-2229-11-109 (PMC3146422; doi:10.1186/1471-2229-11-109)
Supplement: Additional file 7 — Promoter sequence analysis of MtCBF4. The 1000 bp upstream from the translation start site of MtCBF4 was scanned by the PLACE tool for transcriptional factor binding-site analysis. Sites are listed according to their position at the promoter. [file 1471-2229-11-109-S7.PDF]

Promoter sequence analysis of *MtCBF4*

Factor Name	Location	Strand	Signal Sequence
GT1CONSENSUS	2	(-)	GRWAAW
IBOXCORE	3	(-)	GATAA
GATABOX	4	(-)	GATA
CAATBOX1	7	(+)	CAAT
ROOTMOTIFTAPOX1	8	(-)	ATATT
ROOTMOTIFTAPOX1	9	(+)	ATATT
DOFCOREZM	17	(+)	AAAG
NODCON2GM	18	(-)	CTCTT
OSE2ROOTNODULE	18	(-)	CTCTT
POLLEN1LELAT52	25	(+)	AGAAA
DOFCOREZM	27	(+)	AAAG
CACTFTPPCA1	35	(+)	YACT
TBOXATGAPB	36	(+)	ACTTTG
DOFCOREZM	37	(-)	AAAG
RYREPEATLEGUMINBOX	43	(-)	CATGCAY
RYREPEATBNNAPA	44	(-)	CATGCA
GT1CONSENSUS	51	(+)	GRWAAW
ARR1AT	54	(-)	NGATT
TATABOX5	59	(-)	TTATTT
POLASIG1	60	(+)	AATAAA
DOFCOREZM	64	(+)	AAAG
POLLEN1LELAT52	66	(+)	AGAAA
SEF4MOTIFGM7S	75	(+)	RTTTTTR
DOFCOREZM	85	(-)	AAAG
PRECONSCRHSP70A	90	(+)	SCGAYNRNNNNNNNNNNNNNNNHD
PRECONSCRHSP70A	90	(+)	SCGAYNRNNNNNNNNNNNNNNNHD
EBOXBNNAPA	95	(+)	CANNTG
MYCATRD22	95	(+)	CACATG
MYCCONSENSUSAT	95	(+)	CANNTG
EBOXBNNAPA	95	(-)	CANNTG
MYCATERD1	95	(-)	CATGTG
MYCCONSENSUSAT	95	(-)	CANNTG
EBOXBNNAPA	97	(+)	CANNTG
MYCATERD1	97	(+)	CATGTG
MYCCONSENSUSAT	97	(+)	CANNTG
EBOXBNNAPA	97	(-)	CANNTG
MYCATRD22	97	(-)	CACATG
MYCCONSENSUSAT	97	(-)	CANNTG
PREATPRODH	125	(-)	ACTCAT
CAREOSREP1	127	(-)	CAACTC
CURECORECR	132	(+)	GTAC
CURECORECR	132	(-)	GTAC
DPBFCOREDCDC3	148	(-)	ACACNNG
GTGANTG10	155	(+)	GTGA
ARR1AT	156	(+)	NGATT
CAATBOX1	158	(-)	CAAT
ARR1AT	160	(+)	NGATT
CACTFTPPCA1	166	(+)	YACT
TATABOXOSPAL	177	(-)	TATTTAA
ROOTMOTIFTAPOX1	180	(-)	ATATT
EBOXBNNAPA	188	(+)	CANNTG
MYCATERD1	188	(+)	CATGTG
MYCCONSENSUSAT	188	(+)	CANNTG

EBOXBNNAPA	188	(-)	CANNTG
MYCATRD22	188	(-)	CACATG
MYCCONSENSUSAT	188	(-)	CANNTG
GTGANTG10	194	(+)	GTGA
CAREOSREP1	198	(-)	CAACTC
GATABOX	204	(-)	GATA
MYBST1	204	(-)	GGATA
CURECORECR	219	(+)	GTAC
CURECORECR	219	(-)	GTAC
CCAATBOX1	222	(+)	CCAAT
CAATBOX1	223	(+)	CAAT
CACTFTPPCA1	226	(+)	YACT
DOFCOREZM	238	(-)	AAAG
TAAAGSTKST1	238	(-)	TAAAG
POLASIG1	239	(-)	AATAAA
TATABOX5	240	(+)	TTATTT
AUXRETGA1GMGH3	244	(-)	TGACGTAA
ACGTATERD1	246	(+)	ACGT
HEXMOTIFTAH3H4	246	(+)	ACGTCA
ACGTATERD1	246	(-)	ACGT
TGACGTVMAMY	246	(-)	TGACGT
ASF1MOTIFCAMV	247	(-)	TGACG
WRKY71OS	248	(-)	TGAC
GTGANTG10	249	(-)	GTGA
CACTFTPPCA1	250	(+)	YACT
DOFCOREZM	252	(-)	AAAG
SEF3MOTIFGM	263	(+)	AACCCA
CACTFTPPCA1	275	(+)	YACT
TBOXATGAPB	276	(+)	ACTTTG
DOFCOREZM	277	(-)	AAAG
WBBOXPCWRKY1	278	(+)	TTTGACY
WBOXATNPR1	279	(+)	TTGAC
WBOXHVISO1	280	(+)	TGACT
WBOXNTERF3	280	(+)	TGACY
WRKY71OS	280	(+)	TGAC
TBOXATGAPB	282	(+)	ACTTTG
DOFCOREZM	283	(-)	AAAG
WBOXATNPR1	285	(+)	TTGAC
WRKY71OS	286	(+)	TGAC
BIHD1OS	286	(-)	TGTCA
ANAERO1CONSENSUS	290	(+)	AAACAAA
DOFCOREZM	294	(+)	AAAG
NODCON1GM	294	(+)	AAAGAT
OSE1ROOTNODULE	294	(+)	AAAGAT
ARR1AT	296	(+)	NGATT
INRNTPSADB	299	(+)	YTCANTYY
GTGANTG10	300	(-)	GTGA
CACTFTPPCA1	301	(+)	YACT
DOFCOREZM	303	(-)	AAAG
UPRMOTIFIIAT	309	(+)	CCNNNNNNNNNNNNCCACG
CAATBOX1	331	(+)	CAAT
WBOXHVISO1	334	(+)	TGACT
WBOXNTERF3	334	(+)	TGACY
WRKY71OS	334	(+)	TGAC
EECCRCAH1	335	(+)	GANTTNC
PYRIMIDINEBOXOSRAMY1A	341	(+)	CCTTTT

DOFCOREZM	342	(-)	AAAG
ARR1AT	347	(+)	NGATT
CAATBOX1	360	(-)	CAAT
DOFCOREZM	369	(+)	AAAG
POLASIG1	373	(-)	AATAAA
TATABOX5	374	(+)	TTATTT
SEF4MOTIFGM7S	376	(+)	RTTTTTR
MARTBOX	378	(+)	TTWTWTTWTT
POLASIG1	379	(-)	AATAAA
TATABOX5	380	(+)	TTATTT
SEF4MOTIFGM7S	382	(+)	RTTTTTR
POLASIG1	385	(-)	AATAAA
TATABOX5	386	(+)	TTATTT
SEF4MOTIFGM7S	404	(-)	RTTTTTR
TATABOX5	407	(-)	TTATTT
POLASIG3	408	(+)	AATAAT
POLASIG2	411	(+)	AATTAAA
POLASIG3	430	(-)	AATAAT
CACTFTPPCA1	435	(+)	YACT
CPBCSPOR	437	(-)	TATTAG
ROOTMOTIFTAPOX1	439	(-)	ATATT
ROOTMOTIFTAPOX1	440	(+)	ATATT
POLASIG3	451	(-)	AATAAT
TATABOX5	452	(+)	TTATTT
SEF4MOTIFGM7S	454	(+)	RTTTTTR
POLASIG2	457	(-)	AATTAAA
POLASIG1	462	(-)	AATAAA
MARTBOX	463	(+)	TTWTWTTWTT
TATABOX5	463	(+)	TTATTT
CACTFTPPCA1	475	(+)	YACT
DOFCOREZM	486	(+)	AAAG
ACGTABREMOTIFA2OSEM	489	(-)	ACGTGKC
GADOWNAT	489	(-)	ACGTGTC
ABRELATERD1	491	(-)	ACGTG
ACGTATERD1	492	(+)	ACGT
HEXMOTIFTAH3H4	492	(+)	ACGTCA
ACGTATERD1	492	(-)	ACGT
TGACGTVMAMY	492	(-)	TGACGT
ASF1MOTIFCAMV	493	(-)	TGACG
WRKY71OS	494	(-)	TGAC
EBOXBNNAPA	496	(+)	CANNTG
MYCCONSENSUSAT	496	(+)	CANNTG
EBOXBNNAPA	496	(-)	CANNTG
MYCCONSENSUSAT	496	(-)	CANNTG
WBOXHVISO1	503	(-)	TGACT
WBOXNTERF3	503	(-)	TGACY
WRKY71OS	504	(-)	TGAC
GTGANTG10	505	(-)	GTGA
RAV1AAT	514	(+)	CAACA
ANAERO3CONSENSUS	527	(+)	TCATCAC
GTGANTG10	530	(-)	GTGA
CACTFTPPCA1	531	(+)	YACT
WBOXNTERF3	538	(-)	TGACY
WRKY71OS	539	(-)	TGAC
GTGANTG10	540	(-)	GTGA
NODCON2GM	550	(+)	CTCTT

OSE2ROOTNODULE	550	(+)	CTCTT
DOFCOREZM	552	(-)	AAAG
GT1CONSENSUS	553	(-)	GRWAAW
GT1GMSCAM4	553	(-)	GAAAAA
GT1CONSENSUS	554	(-)	GRWAAW
DOFCOREZM	559	(-)	AAAG
POLLEN1LELAT52	560	(-)	AGAAA
SEBFCONSSTPR10A	563	(+)	YTGTCWC
BIHD1OS	564	(+)	TGTCA
WRKY71OS	565	(-)	TGAC
GTGANTG10	566	(-)	GTGA
CACTFTPPCA1	567	(+)	YACT
GATABOX	570	(-)	GATA
WBBOXPCWRKY1	574	(-)	TTTGACY
WBOXHVISO1	574	(-)	TGACT
WBOXNTERF3	574	(-)	TGACY
WBOXATNPR1	575	(-)	TTGAC
WRKY71OS	575	(-)	TGAC
TATABOX5	578	(-)	TTATTT
POLASIG1	579	(+)	AATAAA
SEF4MOTIFGM7S	581	(-)	RTTTTTR
DOFCOREZM	587	(-)	AAAG
POLLEN1LELAT52	588	(-)	AGAAA
IBOXCORENT	590	(-)	GATAAGR
IBOX	591	(-)	GATAAG
SREATMSD	592	(+)	TTATCC
IBOXCORE	592	(-)	GATAA
TATCCAOSAMY	593	(+)	TATCCA
GATABOX	593	(-)	GATA
MYBST1	593	(-)	GGATA
ROOTMOTIFTAPOX1	599	(-)	ATATT
ROOTMOTIFTAPOX1	600	(+)	ATATT
TATABOX3	601	(+)	TATTAAT
TATABOX3	602	(-)	TATTAAT
CACTFTPPCA1	607	(+)	YACT
ROOTMOTIFTAPOX1	611	(+)	ATATT
EECCRCAH1	617	(-)	GANTTNC
ARR1AT	620	(-)	NGATT
SORLIP1AT	632	(+)	GCCAC
ANAERO1CONSENSUS	643	(+)	AAACAAA
DOFCOREZM	647	(+)	AAAG
SORLIP1AT	650	(+)	GCCAC
DPBFCOREDCDC3	653	(+)	ACACNNG
ABRERATCAL	653	(-)	MACGYGB
ABRERATCAL	654	(+)	MACGYGB
CACGTGMOTIF	654	(+)	CACGTG
EBOXBNNAPA	654	(+)	CANNTG
MYCCONSENSUSAT	654	(+)	CANNTG
ABRELATERD1	654	(-)	ACGTG
CACGTGMOTIF	654	(-)	CACGTG
DPBFCOREDCDC3	654	(-)	ACACNNG
EBOXBNNAPA	654	(-)	CANNTG
MYCCONSENSUSAT	654	(-)	CANNTG
ABRELATERD1	655	(+)	ACGTG
ACGTABREMOTIFA2OSEM	655	(+)	ACGTGKC
ACGTATERD1	655	(+)	ACGT

GADOWNAT	655	(+)	ACGTGTC
ACGTATERD1	655	(-)	ACGT
BIHD1OS	658	(+)	TGTCA
TGTCACACMCUCUMISIN	658	(+)	TGTCACA
WRKY71OS	659	(-)	TGAC
GTGANTG10	660	(-)	GTGA
DOFCOREZM	671	(-)	AAAG
POLLEN1LELAT52	672	(-)	AGAAA
WRKY71OS	676	(+)	TGAC
BIHD1OS	676	(-)	TGTCA
NODCON2GM	685	(+)	CTCTT
OSE2ROOTNODULE	685	(+)	CTCTT
DOFCOREZM	687	(-)	AAAG
SEF3MOTIFGM	695	(+)	AACCCA
TATABOX5	701	(-)	TTATTT
POLASIG1	702	(+)	AATAAA
SEF3MOTIFGM	706	(+)	AACCCA
INRNTPSADB	721	(+)	YTCANTYY
CAATBOX1	723	(+)	CAAT
GT1CORE	730	(-)	GGTTAA
MYB1AT	731	(+)	WAACCA
CACTFTPPCA1	738	(-)	YACT
CURECORECR	739	(+)	GTAC
CURECORECR	739	(-)	GTAC
GT1CONSENSUS	751	(+)	GRWAAW
CACTFTPPCA1	757	(+)	YACT
CAATBOX1	777	(-)	CAAT
WBOXATNPR1	778	(+)	TTGAC
WRKY71OS	779	(+)	TGAC
BIHD1OS	779	(-)	TGTCA
CACTFTPPCA1	787	(+)	YACT
ARE1	799	(-)	RGTGACNNNGC
BIHD1OS	803	(+)	TGTCA
WRKY71OS	804	(-)	TGAC
GTGANTG10	805	(-)	GTGA
CACTFTPPCA1	806	(+)	YACT
DOFCOREZM	808	(-)	AAAG
INRNTPSADB	821	(+)	YTCANTYY
GTGANTG10	822	(-)	GTGA
CACTFTPPCA1	823	(+)	YACT
INRNTPSADB	826	(+)	YTCANTYY
GT1CONSENSUS	830	(-)	GRWAAW
GT1GMSCAM4	830	(-)	GAAAAA
GT1CONSENSUS	831	(-)	GRWAAW
DOFCOREZM	840	(-)	AAAG
TAAAGSTKST1	840	(-)	TAAAG
TATAPVTRNALEU	841	(+)	TTTATATA
TATABOX4	842	(-)	TATATAA
SORLREP3AT	844	(-)	TGTATATAT
NODCON1GM	852	(-)	AAAGAT
OSE1ROOTNODULE	852	(-)	AAAGAT
DOFCOREZM	854	(-)	AAAG
CACTFTPPCA1	867	(+)	YACT
DOFCOREZM	869	(-)	AAAG
RAV1AAT	884	(+)	CAACA
SEF4MOTIFGM7S	893	(-)	RTTTTTR

CACTFTPPCA1	900	(+)	YACT
TBOXATGAPB	906	(-)	ACTTTG
DOFCOREZM	907	(+)	AAAG
CACTFTPPCA1	909	(-)	YACT
CURECORECR	910	(+)	GTAC
CURECORECR	910	(-)	GTAC
CACTFTPPCA1	916	(-)	YACT
CURECORECR	917	(+)	GTAC
CURECORECR	917	(-)	GTAC
CACTFTPPCA1	918	(+)	YACT
CACTFTPPCA1	922	(+)	YACT
MYBATRD22	924	(+)	CTAACCA
SV40COREENHAN	924	(-)	GTGGWWHG
MYB1AT	925	(+)	WAACCA
CACTFTPPCA1	931	(+)	YACT
GT1CONSENSUS	936	(-)	GRWAAW
GT1GMSCAM4	936	(-)	GAAAAA
CACTFTPPCA1	946	(-)	YACT
AMYBOX1	956	(+)	TAACARA
GAREAT	956	(+)	TAACAAR
MYBGAHV	956	(+)	TAACAAA
AACACOREOSGLUB1	957	(+)	AACAAAC
DOFCOREZM	963	(-)	AAAG
CACTFTPPCA1	972	(+)	YACT
SP8BFIBSP8BIB	972	(+)	TACTATT
-10PEHVPSBD	975	(+)	TATTCT
DOFCOREZM	979	(-)	AAAG
MARTBOX	980	(+)	TTWTWTTWTT
MARTBOX	981	(+)	TTWTWTTWTT
MARTBOX	982	(+)	TTWTWTTWTT
MARTBOX	983	(+)	TTWTWTTWTT
MARTBOX	984	(+)	TTWTWTTWTT
MARTBOX	985	(+)	TTWTWTTWTT
MARTBOX	986	(+)	TTWTWTTWTT
